# Pemigatinib in previously treated solid tumors with activating *FGFR1–FGFR3* alterations: phase 2 FIGHT-207 basket trial

**DOI:** 10.1038/s41591-024-02934-7

**Published:** 2024-05-06

**Authors:** Jordi Rodón, Silvia Damian, Muhammad Furqan, Jesús García-Donas, Hiroo Imai, Antoine Italiano, Iben Spanggaard, Makoto Ueno, Tomoya Yokota, Maria Luisa Veronese, Natalia Oliveira, Xin Li, Aidan Gilmartin, Michael Schaffer, Lipika Goyal

**Affiliations:** 1 The University of Texas MD Anderson Cancer Center, Houston, TX USA; 2 Fondazione IRCCS Istituto Nazionale dei Tumori, Milan, Italy; 3 University of Iowa, Iowa City, IA USA; 4 Centro Integral Oncologico Clara Campal, Madrid, Spain; 5 Tohoku University Hospital, Sendai-Shi, Japan; 6 Institut Bergonié, Bordeaux, France; 7 Faculty of Medicine, University of Bordeaux, Bordeaux, France; 8 Rigshospitalet Copenhagen University Hospital, Copenhagen, Denmark; 9 Kanagawa Cancer Center, Yokohama, Japan; 10 Shizuoka Cancer Center, Shizuoka, Japan; 11 Incyte International Biosciences Sàrl, Morges, Switzerland; 12 Incyte Corporation, Wilmington, DE USA; 13 Mass General Cancer Center, Harvard Medical School, Boston, MA USA; 14 Stanford Cancer Center, Stanford School of Medicine, Stanford, CA USA

**Keywords:** Targeted therapies, Cancer genetics, Bladder cancer, Bile duct cancer, CNS cancer

## Abstract

Fibroblast growth factor receptor (*FGFR*) alterations drive oncogenesis in multiple tumor types. Here we studied pemigatinib, a selective, potent, oral FGFR1–FGFR3 inhibitor, in the phase 2 FIGHT-207 basket study of *FGFR-*altered advanced solid tumors. Primary end points were objective response rate (ORR) in cohorts A (fusions/rearrangements, *n* = 49) and B (activating non-kinase domain mutations, *n* = 32). Secondary end points were progression-free survival, duration of response and overall survival in cohorts A and B, and safety. Exploratory end points included ORR of cohort C (kinase domain mutations, potentially pathogenic variants of unknown significance, *n* = 26) and analysis of co-alterations associated with resistance and response. ORRs for cohorts A, B and C were 26.5% (13/49), 9.4% (3/32) and 3.8% (1/26), respectively. Tumors with no approved FGFR inhibitors or those with alterations not previously confirmed to be sensitive to FGFR inhibition had objective responses. In cohorts A and B, the median progression-free survival was 4.5 and 3.7 months, median duration of response was 7.8 and 6.9 months and median overall survival was 17.5 and 11.4 months, respectively. Safety was consistent with previous reports. The most common any-grade treatment-emergent adverse events were hyperphosphatemia (84%) and stomatitis (53%). *TP53* co-mutations were associated with lack of response and *BAP1* alterations with higher response rates. *FGFR1*–*FGFR3* gatekeeper and molecular brake mutations led to acquired resistance. New therapeutic areas for FGFR inhibition and drug failure mechanisms were identified across tumor types. ClinicalTrials.gov identifier: NCT03822117.

## Abstract (web summary)

In a tumor-agnostic phase 2 basket trial, the oral *FGFR1*–*FGFR3* inhibitor pemigatinib elicits responses in tumor types beyond cholangiocarcinoma and bladder cancer and in tumor types with rarer FGFR alterations, with insights provided into resistance mechanisms.

## Main

*FGFR* genes harbor pathogenic variants in an array of cancers^1^. Mutations, fusions and amplifications involving *FGFR1–FGFR3* collectively occur in up to 7% of cancers^1–3^. As a key regulator of physiological functions, including cell migration, proliferation and survival, FGFR can drive oncogenesis when its signaling is altered by mutation^1,4^. Thus, FGFR is an attractive drug target, with selective FGFR inhibitors gaining regulatory approval in disease-specific contexts^5–8^.

The *FGFR*-altered tumor types with approved FGFR inhibitors are urothelial cancer, cholangiocarcinoma and myeloid and lymphoid neoplasms (MLNs). In advanced refractory urothelial tract and bladder cancers, where *FGFR3* mutations are frequent^2^, the reversible FGFR1–FGFR4 inhibitor erdafitinib is approved for tumors harboring *FGFR2* or *FGFR3* point mutations or fusions^6^. In advanced refractory cholangiocarcinoma, where *FGFR2* fusions predominate^2^, the reversible FGFR1–FGFR3 inhibitor pemigatinib^5^ and the irreversible FGFR1–FGFR4 inhibitor futibatinib are approved for tumors with *FGFR2* fusions or other rearrangements^7^. In relapsed or refractory MLNs, pemigatinib gained approval for patients with *FGFR1* rearrangements^5^.

Evidence of other potentially oncogenic and actionable *FGFR* alterations and potentially responsive tumors are emerging, providing compelling rationale for evaluating FGFR inhibition in a tumor-agnostic trial. *FGFR1–FGFR3* fusions and point mutations in tumors of different histologies have demonstrated sensitivity to FGFR inhibition in early phase studies, including FIGHT-101, the first-in-human, phase 1 study of pemigatinib^8–16^. Moreover, *FGFR* alterations, including in-frame insertions and truncating deletions, have been described as potential oncogenic drivers but have not been clinically established as actionable^17^. Essential questions remain about the sensitivity of these rarer gene alterations to FGFR inhibition, the sensitivity of different *FGFR*-altered tumor histologies, the impact of specific gene co-alterations on response to FGFR inhibitors and mechanisms of drug failure across histologies.

Given the diversity of *FGFR* alterations and the variety of histologic contexts in which they appear, we sought to evaluate the therapeutic importance of *FGFR* alterations in multiple tumor types. Building on preclinical and phase 1 data^9,13^, the phase 2 FIGHT-207 basket study was designed to evaluate pemigatinib in patients with previously treated unresectable or metastatic solid tumors with *FGFR1–FGFR3* fusions/rearrangements or mutations (NCT03822117; EudraCT, 2018-004768-69). Here we report the clinical outcomes of the study and the biological correlates of intrinsic and acquired resistance from analysis of tissue and circulating tumor DNA (ctDNA) samples.

## Results

### End points

The primary end points were ORR (percentage of patients with complete responses or partial responses) confirmed by independent review committee (IRC) per Response Evaluation in Solid Tumors (RECIST) v.1.1 criteria or Response Assessment in Neuro-Oncology (RANO) in cohorts A and B. Secondary end points were duration of response (DOR), IRC-assessed progression-free survival (PFS), overall survival (OS) and safety and tolerability as assessed by the incidence, type, and severity of adverse events (AEs) in cohorts A and B. Selected exploratory end points were ORR, DOR, PFS and OS in cohort C and genomic analysis of baseline and on-treatment tumor and plasma samples for markers of response and pemigatinib resistance. IRC-assessed clinical benefit rate (CBR) in all cohorts was conducted as a post hoc analysis.

### Patients

**Fig. 1:**
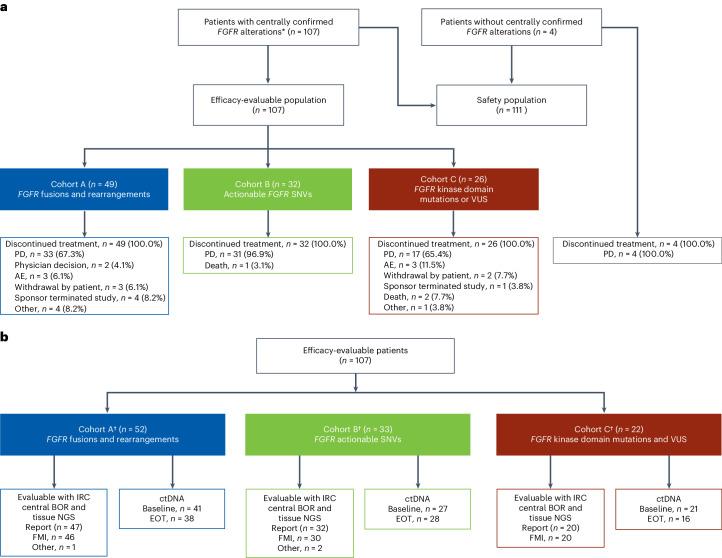
Patient disposition and samples for genomic analysis. **a**, Patient disposition. **b**, Samples for genomic analysis. The primary reason for treatment discontinuation is shown for each patient. *FoundationOne, FMI. ^†^The four patients originally misassigned to cohort C based on local test uncertainty were analyzed here with the relevant set of gene alterations in cohorts A and B. EOT, end of treatment; FMI, Foundation Medicine, Inc.

Between 17 October 2019 and 12 July 2021, 111 patients enrolled. Of these, 107 patients were divided into three cohorts: A (*FGFR1–FGFR3* fusions/rearrangements; *n* = 49), B (activating *FGFR1–FGFR3* non-kinase domain single-nucleotide variants (SNVs); *n* = 32) or C (*FGFR1–FGFR3* kinase domain mutations or variants of unknown significance (VUS) with potential pathogenicity; *n* = 26; Fig. 1a). Four remaining patients were included in the safety analysis but were excluded from the efficacy analysis per protocol because their *FGFR* alterations were not centrally confirmed (Supplementary Table 1). All patients received pemigatinib 13.5 mg orally once daily (QD) continuously. Of the patients in the efficacy-evaluable cohorts, 89 had ctDNA analysis for plasma collected at baseline and, among these, 73 had both baseline and progression samples (Fig. 1b).

Median age among efficacy-evaluable patients was 62 (range, 25–84) years. Overall, 57% of patients were women, 69% were white and 23% were Asian (Table 1). Cholangiocarcinoma (16%), urothelial tract/bladder cancer (11%) and glioblastoma (9.3%) were the most common tumors. Duration of treatment was longest in cohort A (median [range], 4.1 months [0.3–20.2]), followed by cohort B (3.2 months [0.2–15.4]) and cohort C (2.1 months [0.2–18.6]). The most common primary reason for treatment discontinuation was disease progression (77%) and the least common primary reason was AEs (5.4%).

**Table 1:** Patient demographics and baseline clinical characteristics

	Cohort A *FGFR* fusions/ rearrangements (*n* = 49)	Cohort B *FGFR* actionable SNVs (*n* = 32)	Cohort C *FGFR* kinase domain SNVs and VUS (*n* = 26)	Total^a^ (*n* = 107)
Age, median (range), y	61.0 (25–82)	67.5 (45–82)	62.0 (29–84)	62.0 (25–84)
Women, *n* (%)	28 (57.1)	19 (59.4)	14 (53.8)	61 (57.0)
Race, *n* (%)				
White	38 (77.6)	20 (62.5)	16 (61.5)	74 (69.2)
Black/African American	0	0	1 (3.8)	1 (0.9)
Asian	9 (18.4)	9 (28.1)	7 (26.9)	25 (23.4)
Not reported/other^b^	2 (4.1)	3 (9.4)	2 (7.7)	7 (6.5)
ECOG PS, *n* (%)				
0	19 (38.8)	15 (46.9)	9 (34.6)	43 (40.2)
1	29 (59.2)	16 (50.0)	14 (53.8)	59 (55.1)
2	1 (2.0)	1 (3.1)	3 (11.5)	5 (4.7)
Current stage, *n* (%)				
Locally advanced	11 (22.4)	3 (9.4)	3 (11.5)	17 (15.9)
Metastatic	38 (77.6)	29 (90.6)	23 (88.5)	90 (84.1)
Previous radiation, *n* (%)	23 (46.9)	12 (37.5)	13 (50.0)	48 (44.9)
Previous surgery for cancer, *n* (%)	25 (51.0)	19 (59.4)	17 (65.4)	61 (57.0)
Local regional therapy, *n* (%)	2 (4.1)	1 (3.1)	1 (3.8)	4 (3.7)
Previous systemic therapy, *n* (%)	43 (87.8)	29 (90.6)	22 (84.6)	94 (87.9)
1	21 (42.9)	8 (25.0)	5 (19.2)	34 (31.8)
2	13 (26.5)	13 (40.6)	9 (34.6)	35 (32.7)
≥3	9 (18.4)	8 (25.0)	8 (30.8)	25 (23.4)
Solid tumor type, *n* (%)				
Adrenal	0	0	1 (3.8)	1 (0.9)
Anal	0	2 (6.3)	0	2 (1.9)
Breast	0	1 (3.1)	5 (19.2)	6 (5.6)
CNS, other^c^	1 (2.0)	0	2 (7.7)	3 (2.8)
Cervical	2 (4.1)	1 (3.1)	0	3 (2.8)
Cholangiocarcinoma	9 (18.4)	5 (15.6)	3 (11.5)	17 (15.9)
Colorectal	2 (4.1)	0	2 (7.7)	4 (3.7)
Endometrial	1 (2.0)	4 (12.5)	3 (11.5)	8 (7.5)
Esophageal	1 (2.0)	0	0	1 (0.9)
Gallbladder	0	0	1 (3.8)	1 (0.9)
Gastric	1 (2.0)	0	0	1 (0.9)
GE/GE junction	1 (2.0)	0	1 (3.8)	2 (1.9)
Glioblastoma	9 (18.4)	0	1 (3.8)	10 (9.3)
Head and neck	1 (2.0)	1 (3.1)	1 (3.8)	3 (2.8)
Nasopharyngeal	1 (2.0)	0	0	1 (0.9)
NSCLC	6 (12.2)	1 (3.1)	0	7 (6.5)
Ovarian	1 (2.0)	0	0	1 (0.9)
Pancreatic	8 (16.3)	0	0	8 (7.5)
Prostate	1 (2.0)	0	1 (3.8)	2 (1.9)
Renal cell carcinoma	1 (2.0)	1 (3.1)	0	2 (1.9)
Salivary gland	1 (2.0)	0	0	1 (0.9)
Sarcoma	0	0	1 (3.8)	1 (0.9)
Urothelial tract/bladder	1 (2.0)	11 (34.4)	0	12 (11.2)
Uterine sarcoma	0	1 (3.1)	0	1 (0.9)
Other	1 (2.0)	4 (12.5)	4 (15.4)	9 (8.4)

ECOG PS, Eastern Cooperative Oncology Group performance status; GE, gastroesophageal; NSCLC, non-small cell lung cancer.

^a^Excludes four patients whose *FGFR* alteration status could not be confirmed by the central laboratory (cervical, *n* = 1; cholangiocarcinoma, *n* = 1; gallbladder, *n* = 1; other, *n* = 1).

^b^Includes patients identifying as other races and patients with missing or not reported race data.

^c^CNS tumors other than glioblastoma.

### Efficacy

**Fig. 2:**
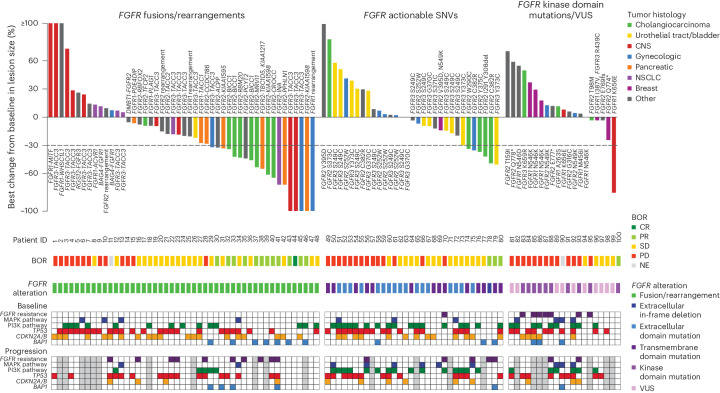
Best percent change from baseline by *FGFR* co-alteration subgroup. Best percent change from baseline by RECIST or RANO for all evaluable patients with tissue NGS report and reported best change in lesion size: *FGFR* fusions/rearrangements (*n* = 48); *FGFR* actionable SNVs (*n* = 32); *FGFR* kinase domain mutations or VUS (*n* = 20). Best OR and PFS by IRC indicated where evaluable. Patients are arranged by *FGFR* alteration type. Bars are colored by major tumor histologies. Dashed lines indicate a criterion for partial response (change from baseline in target lesion size ≥30%). Tumors are grouped into the following histologies based on ≥5 patients: Cholangiocarcinoma, gynecologic cancers (cervical, endometrial and uterine), CNS (glioblastoma, low-grade pediatric glioma and astrocytoma), pancreatic cancer, breast cancer, urothelial tract/bladder cancer, non-small cell lung cancer and other (adrenal cancer, anal cancer, cancer of unknown primary origin, colorectal cancer, gastric/gastroesophageal cancer, gallbladder cancer, giant cell bone tumor, head and neck cancer, lung neuroendocrine cancer, nasopharyngeal cancer, ovarian cancer, prostate cancer, renal cell cancer, sarcoma and solitary fibrous tumor). Genomic analysis is included for all reportable samples and included NGS analysis of tumor tissues and ctDNA at baseline, and of ctDNA at time of progression (gray boxes indicate no report).

**Extended Data Fig. 1:**
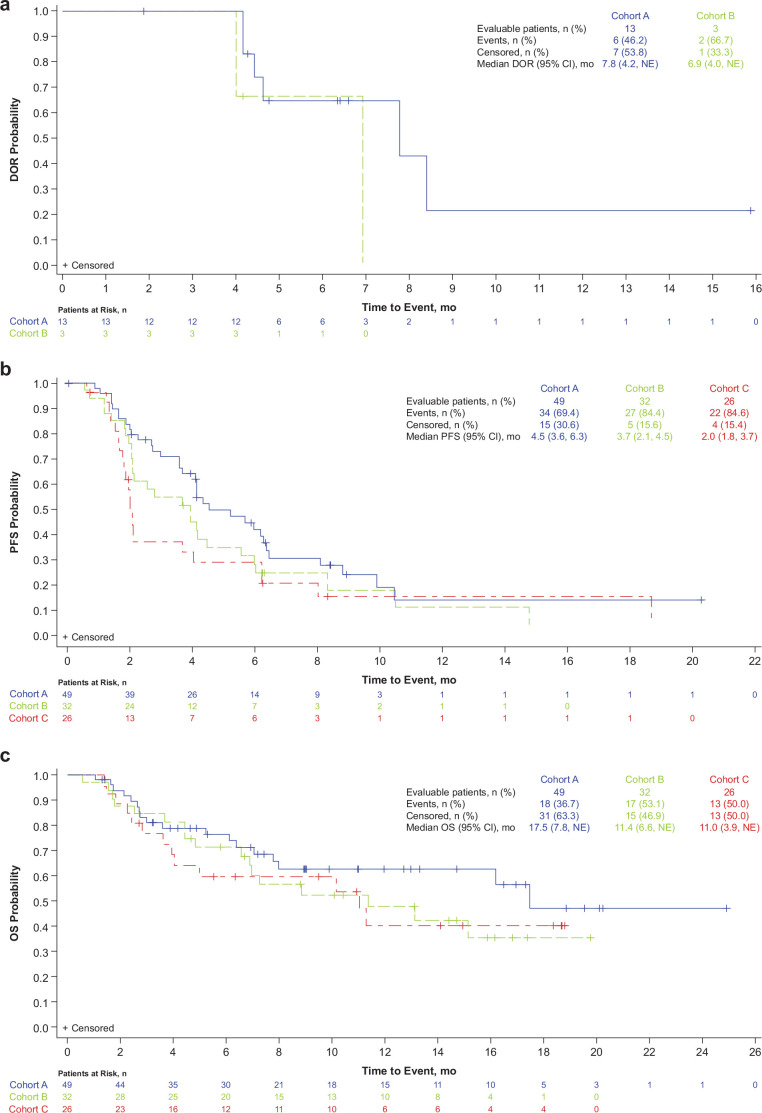
(A) DOR and (B) PFS Based on IRC Assessment per RECIST v1.1 or RANO and (C) OS (Efficacy-Evaluable Population). DOR, duration of response; IRC, independent review committee; NE, not estimable; OS, overall survival; PFS, progression-free survival; RANO, Response Assessment in Neuro-Oncology; RECIST, Response Evaluation Criteria in Solid Tumors.

The primary end points were ORRs in cohorts A and B. ORR (95% confidence interval (CI)) in cohort A was 27% (15%, 41%; *n* = 13) and 9.4% (2%, 25%; *n* = 3) in cohort B. ORR (95% CI) in cohort C, which was an exploratory end point, was 3.8% (0.1%, 20%; *n* = 1; Fig. 2 and Table 2). One patient in cohort A had a complete response. Secondary end points were DOR, PFS and OS in cohorts A and B. Median DOR was 7.8 months in cohort A and 6.9 months in cohort B. Median PFS and OS in cohort A were 4.5 and 17.5 months, respectively, and 3.7 and 11.4 months in cohort B, respectively. Efficacy outcomes are summarized in Table 2 and Extended Data Fig. 1.

**Table 2:** Efficacy outcomes

Parameter	Cohort A *FGFR* fusions/rearrangements (*n* = 49)	Cohort B *FGFR* actionable SNVs (*n* = 32)	Cohort C *FGFR* kinase domain mutations and VUS (*n* = 26)
ORR, % (95% CI)	26.5 (15.0, 41.1)	9.4 (2.0, 25.0)	3.8 (0.1, 19.6)
CBR, % (95% CI)	28.6 (16.6, 43.3)	21.9 (9.3, 40.0)	15.4 (4.4, 34.9)
BOR, *n* (%)			
CR	1 (2.0)	0	0
PR	12 (24.5)	3 (9.4)	1 (3.8)
SD	19 (38.8)	15 (46.9)	8 (30.8)
PD	12 (24.5)	13 (40.6)	15 (57.7)
Not evaluable	4 (8.2)	1 (3.1)	2 (7.7)
Not assessed	1 (2.0)	0	0
DOR, median (95% CI), mo	7.8 (4.2, NE)	6.9 (4.0, NE)	6.2^a^
PFS, median (95% CI), mo	4.5 (3.6, 6.3)	3.7 (2.1, 4.5)	2.0 (1.8, 3.7)
OS, median (95% CI), mo	17.5 (7.8, NE)	11.4 (6.6, NE)	11.0 (3.9, NE)

BOR, best overall response; CR, complete response; NE, not estimable; PD, progressive disease; PR, partial response; SD, stable disease. IRC-confirmed tumor responses were assessed per RECIST or RANO criteria.

^a^Only one patient in cohort C had an objective response; therefore, 95% CI could not be calculated.

**Fig. 3:**
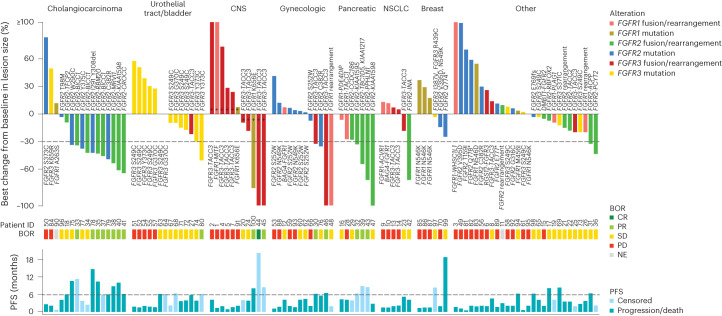
Best percent change from baseline by tumor type. Best percent change from baseline by RECIST or RANO (denoted by +) for all evaluable patients with tissue NGS report and reported best change in lesion size; BOR and PFS by IRC indicated where evaluable. Patients are arranged by major tumor histologies as previously described. Bars are colored by *FGFR* alteration type. Dashed lines indicate a criterion for partial response (change from baseline in target lesion size ≥30%; top) and clinical benefit (PFS ≥ 6 months; bottom). Tumors are grouped into the following histologies based on ≥5 patients: Cholangiocarcinoma, gynecologic cancers (cervical, endometrial and uterine), CNS (glioblastoma, low-grade pediatric glioma and astrocytoma), pancreatic cancer, breast cancer, urothelial tract/bladder cancer, non-small cell lung cancer and other (adrenal cancer, anal cancer, cancer of unknown primary origin, colorectal cancer, gastric/gastroesophageal cancer, gallbladder cancer, giant cell bone tumor, head and neck cancer, lung neuroendocrine cancer, nasopharyngeal cancer, ovarian cancer, prostate cancer, renal cell cancer, sarcoma and solitary fibrous tumor).

Objective responses were observed in multiple tumor types, including histologies for which no FGFR inhibitors are approved (Fig. 3 and Supplementary Table 2). Histologies of particular note included central nervous system (CNS) tumors, pancreatic tumors (all *KRAS* wild-type), cervical tumors and urothelial carcinomas harboring *FGFR* fusions or mutations.

### Safety

Among 111 patients who received ≥1 dose of pemigatinib, no new safety signals were seen. A full list of treatment-emergent AEs (TEAEs) is provided in Supplementary Table 3. The rate of grade ≥3 TEAEs was 68% (Extended Data Table 1). Fatal TEAEs occurred in six patients and included general physical health deterioration (*n* = 3; 2.7%), acute respiratory failure (*n* = 1; 0.9%), confusional state (*n* = 1; 0.9%) and sepsis (*n* = 1; 0.9%). None of the fatal TEAEs was considered by investigators to be related to pemigatinib. TEAEs leading to dose interruption and reduction occurred in 79 (71%) and 48 (43%) patients, respectively. Eight (7.2%) patients discontinued pemigatinib due to TEAEs. The most common any-grade TEAEs were hyperphosphatemia (84%) and stomatitis (53%). Nail toxicities and serous retinal detachment occurred in 45% and 14% of patients.

### Genomic analysis of putative primary driver FGFR alterations

Clinical genomic analysis was performed on tissue and plasma samples collected from patients in cohorts A, B and C. Four patients from cohort C, initially determined with local testing to have VUS, were reassigned for this translational analysis to the other cohorts based on central review and reconsideration of their gene alterations. *DMBT1-FGFR2* (patient 16) and *FGFR1* rearrangements with indeterminate partner (patient 26 and patient 48) were assigned to cohort A and *FGFR3* G370C (patient 57) was assigned to cohort B.

Among the *FGFR* gene alterations, fusions were most sensitive to FGFR inhibition (Fig. 2). The majority of patients in this cohort had type II *FGFR* fusions (*n* = 49; 94%), wherein *FGFR* was the 5′ fusion gene and the breakpoint occurred after the kinase domain in the region spanning intron 17 to exon 18 (ref. ^18^). Three additional rearrangements (*BAG4-FGFR1*, *RGS12-FGFR3* and *DMBT1-FGFR2*) were considered putative type I fusions, a less-common oncogenic *FGFR* rearrangement observed primarily in MLNs, wherein a 5′ partner gene fuses with *FGFR* at a breakpoint after the transmembrane domain^18^. Both type I and II fusions are typically oncogenic and can be sensitive to FGFR inhibition. Although *FGFR* fusions and rearrangements were the most responsive gene alterations across tumor histologies, response was not uniform across histologies; differential rates of objective response and clinical benefit may indicate differential dependencies on FGFR across histologies with common gene alterations subgroups; however, given the relatively small populations evaluated for each histology, analysis of larger populations will likely be required for a more definitive assessment of FGFR pathway dependencies.

*FGFR* non-kinase domain SNVs that were considered actionable based on publicly available alterations databases or clinical study data (cohort B) were localized in extracellular and transmembrane domains. Among these *FGFR* SNVs, clinical benefit was observed for patients with urothelial carcinoma (*n* = 4), cholangiocarcinoma (*n* = 3) and squamous cell carcinoma (*n* = 1). Among five patients with intrahepatic cholangiocarcinoma that had *FGFR2* SNVs, two (C382R (patient 79) and extracellular domain in-frame deletion I291_Y308D del (patient 78)) experienced partial response and two (W290C (patient 75) and Y375C (patient 77)) had stable disease with PFS of 10.5 and 3.7 months, respectively. While cholangiocarcinomas harboring these actionable mutations are less prevalent than *FGFR2* rearrangements, they seem to represent an additional population that may benefit from FGFR inhibition.

*FGFR* kinase domain mutations (cohort C) were considered to be of uncertain actionability given that some kinase domain mutations demonstrate reduced sensitivity to FGFR inhibitors, including pemigatinib in preclinical models^19^. Notably, 2 of 12 patients with *FGFR* kinase domain mutations experienced clinical benefit. One patient with *FGFR1* K656E grade II diffuse astrocytoma had a partial response (patient 100) and one patient with an *FGFR1* N546K low-grade pediatric type glioma had stable disease and a 6.2-month PFS. Notably, activating mutations in K656 in the *FGFR1* activation loop and N546, a controlling residue in the ‘molecular brake’ function, represent the two most common sites of activating *FGFR1* SNVs in gliomas and other CNS tumors; however, among the remaining ten patients with kinase domain mutations without clinical benefit, eight had mutations in molecular brake residues (Extended Data Table 2; *FGFR1* N546K/D (*n* = 5); *FGFR2* N549K (*n* = 3)). Four additional patients in cohort C had mutations downstream of the *FGFR2* kinase domain (patients 82, 89, 98 and 99). These mutations produce truncations before exon 18 and were recently described to be potentially pathogenic^17^. Among these, two patients (Q774* (patient 99) and E769fs (patient 98)) had stable disease ≥6 months, suggesting a modest but real clinical benefit.

Tissue next-generation sequencing (NGS) analysis also identified instances of *FGFR* amplification (defined as *FGFR* copy number ≥6). Concurrent *FGFR* gene amplifications were detected in nine patients (Supplementary Table 4), including concurrent amplifications with the corresponding *FGFR* mutation (*n* = 4) or *FGFR* fusion/rearrangement (*n* = 1) as well as *FGFR* amplifications occurring in an alternative *FGFR* to the enrollable *FGFR* gene alteration (*n* = 4). There were not enough patients in FIGHT-207 with concurrent *FGFR* gene amplification to conclude whether it had a meaningful impact on response to pemigatinib.

### Correlation of co-alterations with patient outcomes

This FIGHT-207 basket study provided the opportunity to assess possible patterns of intrinsic resistance associated with co-alterations across multiple histologies and multiple *FGFR* alterations using combined genomic analysis of tumor tissue and ctDNA. Among patients with *FGFR* fusions/rearrangements and actionable SNVs (cohorts A and B, respectively), 79 evaluable patients had baseline tissue sequencing and 55 of these additionally had baseline ctDNA sequencing. Baseline ctDNA analysis had limited concordance with tissue NGS analysis for detection of *FGFR* variants and some co-alterations across all study samples (Supplementary Fig. 1), likely explained by multiple technical (for example, assay sensitivity, analytical thresholds for variant reporting and variable variant annotations) and biological (for example, age of samples and variable ctDNA shedding) factors. This correlation analysis is therefore focused on the complementary value of combining the gene alterations detectable by the two methods. Tumors were categorized as having a specific co-mutation if this mutation was seen by tissue or ctDNA analysis or both. Based on baseline tissue NGS analysis alone, patterns seen in patients with *FGFR2* fusion-positive cholangiocarcinoma in FIGHT-202 were recapitulated here across multiple histologies harboring a variety of *FGFR1–FGFR3* fusions and mutations. Specifically, none of 27 patients with tumors harboring alterations in *TP53* had an objective response. Moreover, patients with tumors with *TP53* alterations or one of several other tumor-suppressor genes had a lower PFS than those with wild-type copies of these genes (Extended Data Table 3). New correlations seen in FIGHT-207 included the associations with oncogenic alterations in the MAPK pathway or inactivating alterations in *ARID1A* with low PFS and between alterations in *BAP1* and high clinical benefit. Notably, by baseline ctDNA analysis alone, these associations with *ARID1A*, MAPK pathway and *BAP1* alterations held, but the association seen with *TP53* and tumor-suppressor gene alterations did not (Extended Data Tables 4–6).

### Acquired resistance in multiple histologies

All 73 patients who had post-progression ctDNA samples with matched baseline ctDNA also had baseline tumor biopsy molecular profiling. Fourteen (19%) patients acquired one or more secondary *FGFR* mutation in the kinase domain, in residues known or likely to confer resistance (Extended Data Table 7)^20–25^. For patients with cholangiocarcinoma, kinase domain mutations emerged exclusively in patients with clinical benefit from pemigatinib, supporting the case for acquired-resistance mechanisms. While diverse *FGFR1–FGFR3* alterations and multiple tumor types were represented, the common pattern across histologies was the emergence of mutations in the gatekeeper residues (*FGFR2* V564F/I/L; *FGFR3* V555L/M) or closely neighboring residues (*FGFR1* V559L/M) and molecular brake residues (*FGFR1* N546K; *FGFR2* N549D/H/K, E565A and K641R). Other emergent *FGFR2* mutations included M537I, L617V and K659M. Ten of 14 (71%) patients developed polyclonal *FGFR* resistance mutations, with most patients developing concurrent gatekeeper and molecular brake residue mutations and many developing co-occurring mutations at the same codon (N549K and N549D). No mutations in an *FGFR* gene other than the originally altered *FGFR* gene were detected in post-progression plasma samples (for example, *FGFR2* mutations were not detected in *FGFR1*-altered tumors).

**Extended Data Fig. 2:**
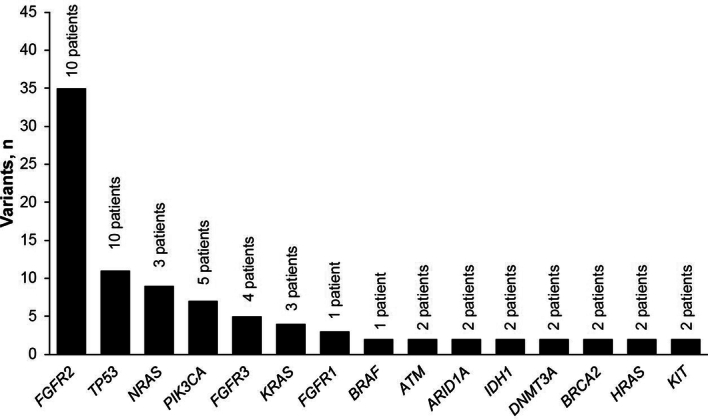
Genes With Most Frequent Emergent Pathogenic or Resistance Variants at Progression by ctDNA. Genes with pathogenic or known resistance variants detected by ctDNA at progression but not at baseline are plotted by number of emergent variants. ctDNA, circulating tumor DNA.

**Extended Data Fig. 3:**
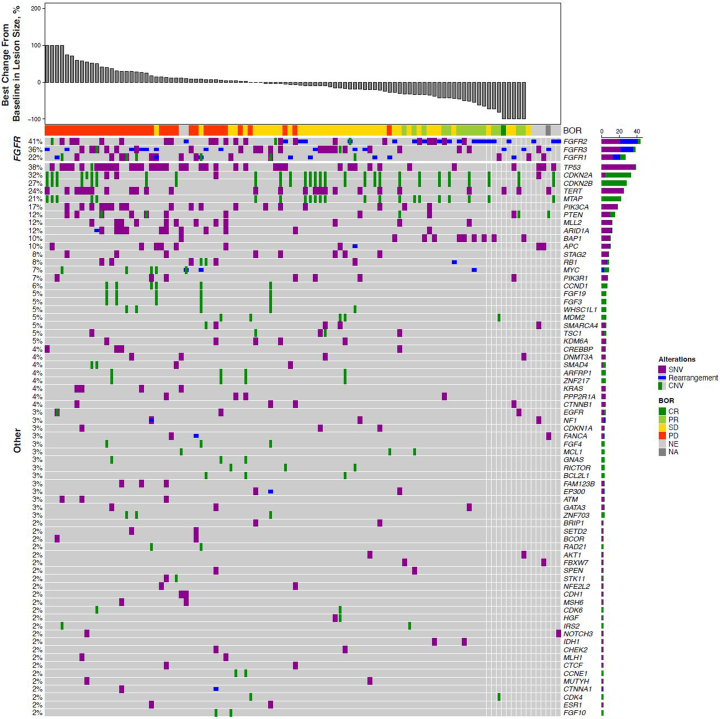
Across-Indication Analysis of Baseline Co-alterations. Analysis of tumor tissue samples includes all evaluable patients from cohorts A, B, and C and central tissue next-generation sequencing (Foundation Medicine, Inc.) reporting. Known or likely pathogenic somatic gene alterations occurring in ≥2% of patients are shown. Patients are arranged by best percent change from baseline per RECIST or RANO. BOR, best overall response; cnv, copy number variation; CR, complete response; FGFR, fibroblast growth factor receptor; IRC, independent review committee; NA, not applicable; NE, not evaluable; PChg, percent change from baseline; PD, progressive disease; PR, partial response; SD, stable disease; snv, single-nucleotide variant.

In addition to secondary *FGFR* variants, new mutations in co-altered genes emerged in end-of-treatment but not baseline plasma ctDNA samples that may be associated with resistance as they involved *TP53, PIK3CA* and/or *RAS* (Extended Data Fig. 2)^26,27^. A larger set of additional emergent variants is presented in Extended Data Fig. 3.

### Pooled co-alteration data from pemigatinib studies

To increase the power of our analysis, we investigated pooling the FIGHT-207 data with datasets from previous pemigatinib clinical studies, including FIGHT-101 (ref. ^9^) (phase 1/2; multiple histologies), FIGHT-201 (ref. ^28^) (phase 2; urothelial tract/bladder cancer) and FIGHT-202 (ref. ^26^) (phase 2; cholangiocarcinoma) in which co-alteration analysis has been previously reported. This analysis included patients with available tissue NGS analysis, *FGFR* fusions/rearrangements or actionable *FGFR* SNVs, centrally determined best overall response and treatment with pemigatinib at or above the recommended dose. Combined FIGHT-101 (*n* = 20) and FIGHT-207 (*n* = 72) data increased the power of the analysis for various solid tumors, but did not result in any change to the identification of co-altered genes significantly correlated with best overall response to pemigatinib. The tumor suppressors *BAP1* and *TP53* remained the genes whose alteration correlated significantly with objective response (Supplementary Table 5). Similarly, analysis of combined FIGHT-202 (*n* = 104) and FIGHT-207 (*n* = 11) data for patients with cholangiocarcinoma (Supplementary Table 6) did not result in any change to the identification of co-altered genes significantly correlated with best overall response to pemigatinib, and only *TP53* was found to be nominally significant (significance was not maintained following stringency correction for multiple testing). Combined FIGHT-201 (*n* = 149) and FIGHT-207 (*n* = 13) data for patients with urothelial carcinoma (Supplementary Table 7) identified *TSC1*, which was reported in earlier analysis and *CDKN1A*, which was now found to be correlated nominally significantly with objective response. Notably, a combined analysis including samples from all four studies was not considered to be valid due to skewing resulting from the inclusion of larger sample sets for cholangiocarcinoma and urothelial carcinoma. This imbalance precludes inference of global correlations of co-alterations with response to pemigatinib.

## Discussion

Oncogenic *FGFR1–FGFR3* alterations are diverse in genomic structural changes, localization and functional consequences^1^. Although clinically validated only in cholangiocarcinoma and bladder cancer, *FGFR* alterations are present in multiple histologies^2^. Basket trials such as FIGHT-207 and the recently completed phase 1 basket study of futibatinib and the phase 2 RAGNAR basket study of erdafitinib offer growing evidence for expanding indications that seem to be actionable with FGFR inhibitors^8,10^. We report not only the safety and efficacy of pemigatinib in this exploratory phase 2 basket study, but leverage the depth of translational data collected in FIGHT-207 to provide five key insights into the biology of FGFR inhibition and the clinical utility of FGFR inhibitors.

First, we observed antitumor activity in cancers beyond cholangiocarcinoma and bladder cancer. Pemigatinib demonstrated activity in patients with CNS tumors, pancreatic cancer and cervical cancer. Similarly, clinical activity in multiple tumor types has been previously reported in other FGFR inhibitor studies^8,10,12,29,30^. While actionable *FGFR* alterations in these cancers are rare (<6%)^2,3^, the benefit of FGFR inhibition seen in this study highlights the value of routine comprehensive molecular screening in solid tumors.

Second, in addition to confirming previous reports that *FGFR2* fusions and other rearrangements in cholangiocarcinoma are sensitive to FGFR inhibition^10,12,30,31^, this study showed in a dedicated cohort of *FGFR*-mutated tumors that specific *FGFR2* SNVs, namely C382R and in-frame deletions, are associated with response to pemigatinib, suggesting that FGFR inhibitors may be effective in cholangiocarcinoma with *FGFR2* alterations other than fusions and rearrangements.

**Fig. 4:**
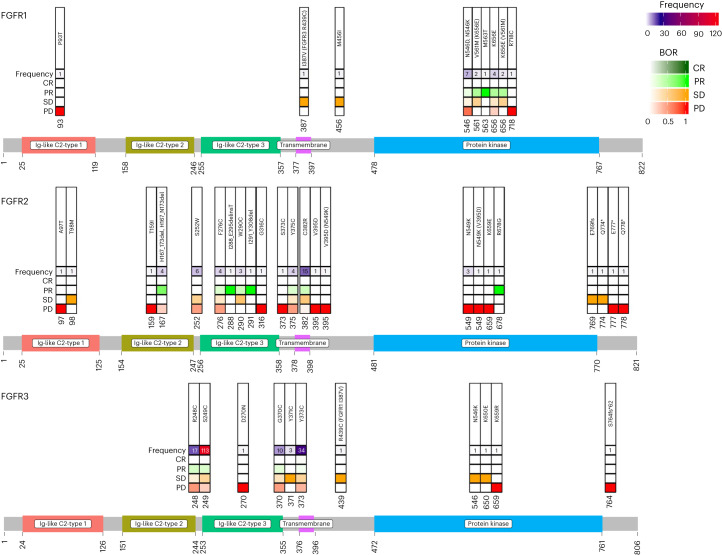
Compilation of *FGFR1–FGFR3* SNVs and associated clinical responses to FGFR inhibitors. Clinical response data for patients with alternative *FGFR1–FGFR3* SNVs treated with pemigatinib (FIGHT-101 (*n* = 9)^9^, FIGHT-201 (*n* = 154)^28^, FIGHT-202 (*n* = 5)^31^, FIGHT-207 (*n* = 53)), futibatinib (*n* = 6)^10^, infigratinib (*n* = 5)^37,38^, Debio1347 (*n* = 5)^32,39^ or RLY-4008 (*n* = 14)^16^ are compiled by site of mutation with indicated rates of BOR. For cases with multiple *FGFR* co-mutations, additional mutations are noted in parentheses. Ig, immunoglobulin.

Third, the dedicated cohort for activating *FGFR2* mutations allowed us to explore the sensitivity of previously clinically unvalidated classes of mutations. In-frame deletions are consistently associated with objective responses. Exon 18 truncating mutations are associated with prolonged stable disease in some instances^32^. In general, de novo *FGFR* kinase domain mutations showed low response to pemigatinib, which was not unexpected as secondary mutations in the kinase domain represent a mechanism of acquired resistance^21,22,24,33–36^; however, we note that exceptional cases of clinical benefit did occur, including one patient with *FGFR1* K656E and one patient with molecular brake mutation *FGFR1* N546K. To systematically characterize the sensitivity of a diverse array of *FGFR1–FGFR3* SNVs to FGFR inhibition in the clinic, we compiled available data from these patients from multiple FGFR inhibitor trials. We reviewed response data for 254 patients with *FGFR1–FGFR3* SNVs treated with at least one of five FGFR inhibitors: pemigatinib (FIGHT-101 (ref. ^9^), FIGHT-201 (ref. ^28^), FIGHT-202 (ref. ^31^) and FIGHT-207), futibatinib^10^, infigratinib^37,38^, Debio1347 (refs. ^32,39^) or RLY-4008 (ref. ^16^) (Fig. 4). The resulting maps indicate that certain activating *FGFR1–FGFR3* SNVs show repeated evidence of clinical benefit in response to FGFR inhibition, providing a rationale for clinical development for these patients.

Fourth, study of potential mechanisms of primary resistance to pemigatinib revealed that baseline co-alterations in tumor suppressors, particularly *TP53* and *ARID1A*, and oncogenic co-alterations in the MAPK pathway were associated with shorter PFS compared to those without alterations. Notably, consistent with data seen in FIGHT-202 where none of nine patients with cholangiocarcinoma and concurrent *TP53* mutations showed an objective response^26^, in FIGHT-207 none of 27 *FGFR*-altered tumors of various histologies with concurrent *TP53* mutations detected in tumor tissue showed an objective response to pemigatinib. Similarly, *TP53* co-alterations were associated with lower ORRs in a cohort of patients with urothelial carcinoma and *FGFR3* alterations treated with erdafitinib under real-world conditions^40^; however, in the FIGHT-201 study in *FGFR-*altered bladder cancer^28^, baseline concurrent *TP53* alterations did not correlate with response or nonresponse to pemigatinib, cautioning against overgeneralization of subgroup analyses. A positive correlation was seen between alterations in *BAP1* and both clinical benefit from and response to pemigatinib. *FGFR2* and *BAP1* alterations commonly co-occur in intrahepatic cholangiocarcinoma^41^, suggesting that the *FGFR2* and *BAP1* co-alteration may represent a distinct cooperative molecular etiology for some cancers. Overall, further prospective studies are needed to validate the correlations seen in this study to assess whether co-mutation status can inform patient selection.

Fifth, serial ctDNA analysis revealed mechanisms of acquired resistance to pemigatinib in a variety of tumor types. To date, our knowledge of acquired resistance to FGFR inhibitors has largely been restricted to *FGFR2* fusion-positive cholangiocarcinoma^22,24,33–36^ and *FGFR3-*altered urothelial cancer^21,25,40^. In our study, patient 16 with advanced pancreatic cancer harboring a *FGFR1–PDE4DIP* fusion developed newly detected mutations in a residue near the gatekeeper (*FGFR1* V559L/M) and in a molecular brake residue (*FGFR1* N546K), standing as the first report of clinical on-target resistance to an FGFR inhibitor in an *FGFR1*-altered tumor or in pancreatic cancer to our knowledge. Consistent with laboratory characterization of acquired *FGFR2* and *FGFR3* resistance mutations in patients with cholangiocarcinoma and urothelial carcinoma, respectively^21,24^, our study also revealed that across *FGFR1–FGFR3*, the most common sites for progression-emergent kinase domain mutations are the gatekeeper residues and the molecular brake residues. Mutations in the gatekeeper residue sterically hinder pemigatinib from binding the receptor^23^, and mutations in the molecular brake residues result in functional gain and conformational shifts that disfavor inhibitor binding^20,23^. Polyclonal resistance with multiple mutations emerging at progression in the same patient was common in our study, as has previously been observed in cholangiocarcinoma but less commonly in urothelial carcinoma^21,22,25^. In addition to patients with cholangiocarcinoma, we saw polyclonal acquired resistance in patients with *FGFR2*-altered gastroesophageal/gastroesophageal junction cancer and cancer of unknown primary origin, *FGFR3-*altered non-small cell lung cancer and *FGFR1-*altered pancreatic cancer. Notably, several next-generation FGFR inhibitors have shown preclinical activity and preliminary clinical activity in patients with cholangiocarcinoma harboring *FGFR2* kinase domain mutations and urothelial cancer harboring *FGFR3* kinase domain mutations following previous FGFR inhibitor treatment^16,21,32,42–44^.

Besides the observed secondary mutations in *FGFRs*, molecular analysis of ctDNA at the time of progression identified other emergent gene variants that may contribute to acquired resistance (on-pathway resistance mutations). Genes with emergent variants were *PIK3CA* and RAS family genes (*KRAS*, *NRAS* and *HRAS*), presumably conferring alternatives for downstream pathway activation. In cholangiocarcinoma, *FGFR2* fusions are generally mutually exclusive with alterations in MAPK pathway (*KRAS*, *NRAS* and *BRAF*) in baseline samples^26^, reflecting their roles as alternative oncogenic drivers. Notably, among the eight evaluable patients with pancreatic tumors in FIGHT-207, seven patients had *FGFR* fusions in the context of the *KRAS* wild-type background, highlighting the importance of testing for *FGFR2* fusions in this population with few therapeutic options. Emergent *PIK3CA* and RAS family mutations were also found to co-occur with acquired *FGFR2* resistance mutations in some patients with cholangiocarcinoma^24^. Co-alterations in PI3K and RAS pathways have similarly been described as conferring bypass resistance in nonclinical models for other FGFR inhibitors^21,24^. The interplay between oncogenic *FGFR1–FGFR3* alterations, acquired on-target resistance mutations and emergent co-alterations compensating for FGFR inhibition requires further study and clinical validation.

One inherent limitation of the basket study design is that heterogeneous tumors and genetic alterations were included, some of which were not well represented. While tumor heterogeneity was intentional by design and a strength for signal finding, the study was terminated early by the sponsor for business reasons and some tumor and molecular cohorts, cohorts A and B, specifically, were therefore underpowered to definitively conclude questions of FGFR dependency for specific alterations and tumor types. The observations of response in this study are nevertheless valuable as indicators for potentially actionable *FGFR* alterations and tumors that warrant deeper investigation. Additionally, heavily pretreated patients enrolled in FIGHT-207 may have had more co-alterations that impacted response. Our study was not designed to evaluate whether the co-alterations we found to be associated with response and PFS were predictive of tumor response to pemigatinib. Interpreting these findings should be carried out with caution, as the association between co-alterations and outcomes may only be prognostic in nature. Finally, it should be noted that safety in this basket study is consistent with what was previously reported in patients with either cholangiocarcinoma or urothelial carcinoma treated with pemigatinib in the FIGHT-202 (ref. ^31^) and FIGHT-201 (ref. ^28^) studies.

In conclusion, we evaluated the clinical activity of pemigatinib in this phase 2 basket study comprising multiple tumor types and including previously untested *FGFR1–FGFR3* alterations. We identified new therapeutic areas for FGFR inhibition in this study and ascertained the highest-sensitivity *FGFR* mutations from a compilation of studies, such that this curated list of mutations can be considered for eligibility in future FGFR inhibitor trials. We also discovered aspects of FGFR biology that transcend observations in cholangiocarcinoma and urothelial cancers and highlight the value of testing for *FGFR* alterations in multiple tumor types. Future work to predict response to pemigatinib is needed to better identify patients with cancer who might benefit from FGFR inhibitor therapy.

## Methods

### Study design

This open-label, single-arm, multicenter phase 2 study consisted of three cohorts defined by *FGFR* alteration category. Patients with in-frame *FGFR1–FGFR3* fusions and rearrangements, including intact kinase domains, were assigned to cohort A. Cohort B consisted of patients with *FGFR* actionable SNVs, excluding kinase domain SNVs, considered known or likely to be activating and actionable. This set included specific somatic missense mutations, insertions or deletions of *FGFR1–FGFR3* that were known or likely activating (based on clinical trial data and public alterations annotations by OncoKB, ClinVar and Omim)^45–47^. Cohort C included the remaining patients with *FGFR1–FGFR3* mutations in the kinase domain or *FGFR1–3* VUS with potential pathogenicity (Fig. 1). Patient enrollment and initial cohort assignment based on genomic or fluorescence in situ hybridization testing results from a local laboratory were permitted. Most patients had local testing using the FoundationOne CDx assay (Foundation Medicine), which detects genomic alterations in 324 genes (>500× median coverage for target genes)^48^. Additional local tests were performed by Caris, Tempus, Guardant360, Oncomine, Riken Genesis Oncoguard and Sophia Genetics laboratories.

Sex and/or gender were not considered in the study design or statistical analysis plan because *FGFR* alterations across histologies have not been shown consistently to predominate in one sex^2^. Moreover, the sex distribution in our study is similar to that of other basket studies of FGFR inhibitors^8,10^. Patients were recruited into FIGHT-207 irrespective of sex or gender. The sex of patients was self-reported, and gender was not collected.

The study was performed in accordance with the International Council for Harmonisation Good Clinical Practice, the principles embodied by the Declaration of Helsinki and local regulatory requirements. The study protocol was approved by the institutional review board of each study site before patient enrollment. All patients provided written informed consent before screening. The sponsor provided medical monitoring of the study, but no data safety monitoring board was established. A full list of investigators and study sites is provided in Supplementary Table 8. The study was terminated by the sponsor for business reasons.

### Patients

Eligible patients were ≥18 years old with a histologically or cytologically confirmed advanced/metastatic or surgically unresectable solid tumor and radiographically measurable disease per RECIST v.1.1 or RANO criteria. Patients were required to have a documented *FGFR1–FGFR3* mutation or fusion/rearrangement, disease progression after ≥1 line of previous systemic therapy, no therapy available likely to provide clinical benefit, ECOG PS ≤2, a baseline tumor specimen and willingness to avoid pregnancy or fathering children.

Exclusion criteria were previous receipt of a selective FGFR inhibitor; concurrent administration or receipt of anticancer medications ≤28 days before first pemigatinib dose; candidacy for potentially curative surgery; clinically notable corneal or retinal disorder confirmed by ophthalmologic examination; current evidence of ectopic mineralization or calcification; radiation administered ≤2 weeks before the first dose of pemigatinib or inadequate recovery from radiation-related toxicities; untreated CNS metastases or CNS metastases that have progressed; additional malignancy requiring active treatment or that is progressing, except for basal cell carcinoma of the skin, squamous cell carcinoma of the skin or in situ cervical cancer that has undergone potentially curative therapy; gastrointestinal disorders that could interfere with the absorption, metabolism or excretion of pemigatinib; inability to swallow and retain oral medication; clinically notable or uncontrolled cardiac disease, except for patients with a pacemaker or well-controlled atrial fibrillation; history or presence of clinically meaningful abnormal electrocardiogram; active chronic or current infectious disease requiring systemic antibiotic, antifungal or antiviral treatment ≤2 weeks before enrollment; active hepatitis B or hepatitis C infections; HIV infection; use of potent cytochrome P450 3A4 (CYP3A4) inhibitors or inducers or moderate CYP3A4 inducers ≤14 days or ≤5 half-lives, whichever is longer, before the first dose of pemigatinib; known hypersensitivity or severe reaction to pemigatinib or its excipients; inadequate recovery from toxicity or complications from major surgery; pregnancy or breastfeeding; receipt of an investigational drug for any indication; history of hypovitaminosis D requiring supraphysiologic doses to correct the deficiency; inability or unlikeliness of the patient to comply with the dose schedule and evaluations; any condition that in the investigator’s opinion may interfere with the full participation in the study, pose a notable risk to the patient or interfere with data interpretation; and inability of the patient to provide informed consent. Patients with laboratory values outside of normal ranges were also excluded. Nonpermitted hematology values were platelets ≤75 × 10^9^ l^−1^, hemoglobin ≤9.0 g dl^−1^ or absolute neutrophil count ≤1.5 × 10^9^ l^−1^. Transfusions were allowed with a 2-week washout period. Laboratory values suggesting hepatic dysfunction were alanine aminotransferase ≥3 × upper limit of normal (ULN; >5 × ULN for liver metastasis), aspartate aminotransferase ≥3 × ULN (>5 × ULN for liver metastasis), total bilirubin ≥1.5 × ULN (≥2.5 × ULN if Gilbert’s syndrome or liver metastasis) or alkaline phosphatase ≥3 × ULN. Prohibited renal values were serum creatinine clearance ≤30 ml min^−1^ based on the Cockcroft–Gault formula. Patients with serum phosphate >ULN or serum calcium outside of normal range or serum albumin-corrected calcium outside of the normal range when serum albumin is outside of the normal range were also excluded.

### Treatment

Patients self-administered pemigatinib on a continuous basis at a starting oral dose of 13.5 mg QD in 21-day cycles until documented radiological disease progression, unacceptable toxicity, withdrawal of consent or physician decision.

### End points and assessments

The primary end points were ORRs in cohorts A and B as determined by IRC. ORR was defined as the percentage of patients who achieved complete response or partial response per RECIST v.1.1 or RANO criteria. Disease was assessed by computed tomography or magnetic resonance imaging at baseline, every three cycles and at the end of treatment.

Secondary end points were IRC-assessed PFS (time from first dose to progressive disease or death, whichever is first) in cohorts A and B, respectively, DOR (time from the first assessment of complete response or partial response until progressive disease or death, whichever is first) in cohorts A and B, respectively, OS (time from first dose to death) in cohorts A and B, respectively, and safety and tolerability as assessed by the incidence and severity of TEAEs and treatment-related AEs according to the National Cancer Institute Common Terminology Criteria for Adverse Events v.5.0.

Selected exploratory end points included ORR, PFS, OS and DOR in cohort C, and baseline and on-treatment tumor and plasma genomic analysis associated with response and resistance.

IRC-assessed CBR (percentage of patients with CR, PR or SD ≥6 months) was also calculated for all cohorts as a post hoc analysis.

### Statistical analyses

Approximately 60 and 90 patients were planned for cohorts A and B, respectively. Assuming ORRs of 35% in cohort A and 30% in cohort B, respectively, 60 and 90 patients were needed to ensure ≥90% power to reject the null hypothesis of ORR ≤ 15% with a one-sided test at the overall 0.025 level of significance. In cohort C, ≈20 patients were enrolled to provide ≥80% chance of observing at least four responders if the underlying ORR was 30%.

The efficacy population included all enrolled patients (*n* = 107) in cohorts A, B and C with *FGFR* alterations confirmed based on genomic testing results from the Foundation Medicine central laboratory who received ≥1 pemigatinib dose. The safety population included all enrolled patients who received ≥1 pemigatinib dose. The primary analysis of ORR in efficacy-evaluable patients in cohorts A and B was based on IRC-confirmed tumor responses, with 95% CI for ORR in all cohorts estimated using the Clopper–Pearson method. PFS, DOR and OS in efficacy-evaluable patients in all cohorts were analyzed with the Kaplan–Meier method; 95% CI for median PFS, DOR and OS were calculated using the generalization of Brookmeyer and Crowley’s method with log–log transformation. The exact 95% CI for the CBR in all cohorts was calculated. Data analyses were performed according to the statistical analysis plan using SAS v.9.4.

### Translational analyses

Genomic data for baseline tissue included all evaluable patients (*n* = 107). Genomic data for plasma ctDNA data from baseline (*n* = 89) and paired at disease progression (*n* = 73) included all available samples from efficacy-evaluable patients. For available samples, PredicineCARE^49^ (Predicine) NGS analysis of plasma cell-free DNA was conducted for 152 genes (approximately 20,000× coverage for target genes) at baseline and at disease progression. Analysis focused on gene alterations, including SNVs, copy-number variants or rearrangements considered to be known or likely pathogenic based on the Foundation Medicine database and incorporating COSMIC status. Analysis of the gene co-alterations correlation with ORR or CBR used Fisher’s exact test, two-sided and correlation with PFS used a log-rank test. Analysis of genes with emergent pathogenic variants at progression included all genes with variants detected in ctDNA exclusively at progression. Translational data analyses were performed in R v.4.1.1.

### Key protocol amendments

#### Amendment 3 (current version): February 2021

In the current version of the protocol, cohort definitions were further refined based on evolving terminology and to clarify which alterations were accepted for cohorts A and C. The current version includes other updates regarding tumor biopsy timing, COVID-19 pandemic mitigation strategies and regulatory requirements in Japan. This version of the full study protocol with confidential information redacted is included in the Supplementary Information supporting the article.

#### Amendment 2: January 2020

Cohort definitions were updated and details of the efficacy analysis were clarified. Other changes were made to incorporate US Food and Drug Administration review feedback received for other pemigatinib study protocols.

#### Amendment 1: February 2019

The protocol was amended to clarify the cohort assignment for patients with unknown fusion partners. Cohort A alterations were updated to include *FGFR2* intron 17 rearrangements and cohort C to include *FGFR1* and *FGFR3* rearrangements with unknown fusion partners. Other revisions were made to incorporate updated safety information and Voluntary Harmonisation Procedure review feedback received for other pemigatinib study protocols.

### Reporting summary

Further information on research design is available in the Nature Portfolio Reporting Summary linked to this article.

## Online content

Any methods, additional references, Nature Portfolio reporting summaries, source data, extended data, supplementary information, acknowledgements, peer review information; details of author contributions and competing interests; and statements of data and code availability are available at 10.1038/s41591-024-02934-7.

### Supplementary information

Supplementary Information Supplementary Tables 1–8 and Fig. 1.

Reporting Summary

## Figures and Tables

**Extended Data Table 1:**
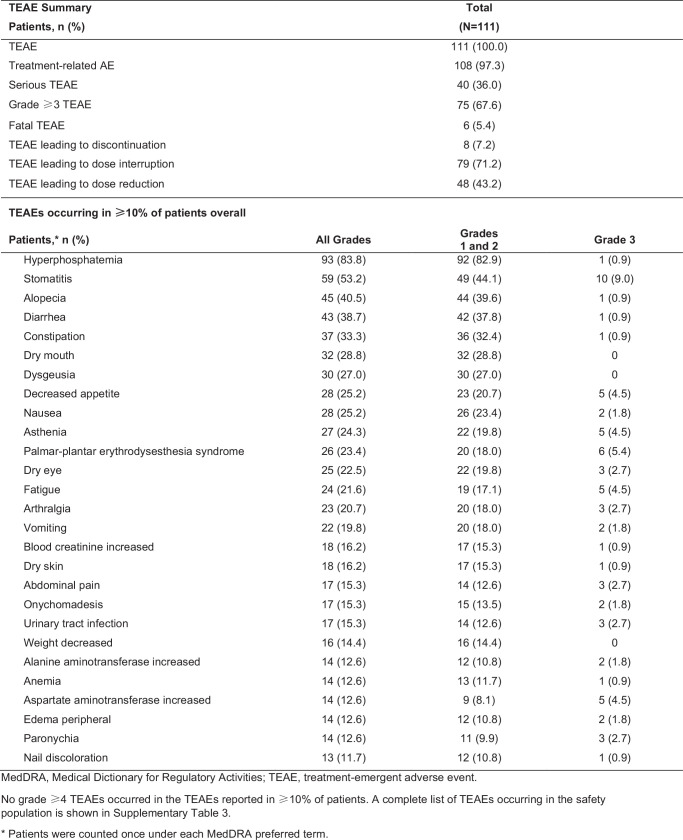
Summary of Treatment-Emergent Adverse Events

**Extended Data Table 2:**
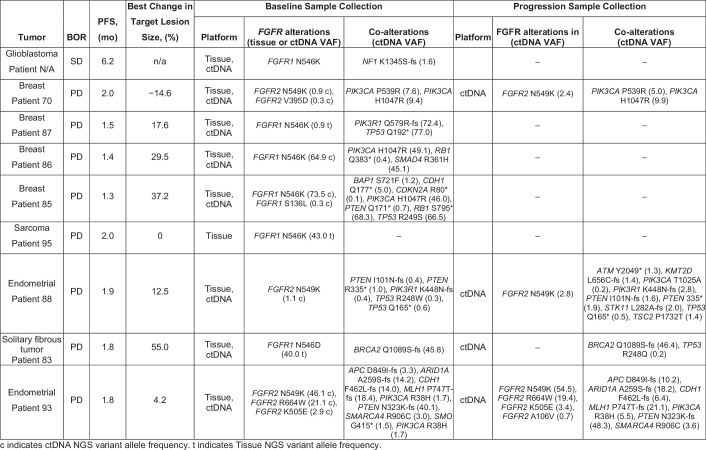
De novo *FGFR* Molecular Brake Mutations in Solid Tumors

**Extended Data Table 3:**
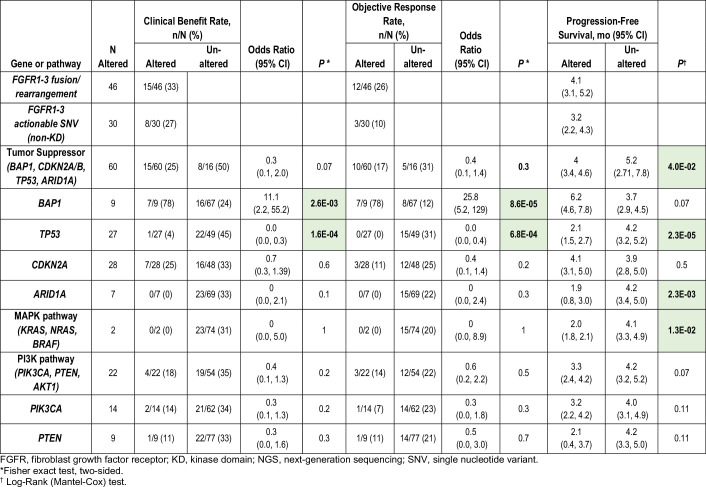
Baseline Co-alterations of Genes Belonging to Select Pathways in Patients with *FGFR* Fusions/ Rearrangements (Cohort A) and *FGFR* Actionable SNVs (Cohort B) Associated With Response (Tissue NGS only; N = 76)

**Extended Data Table 4:**
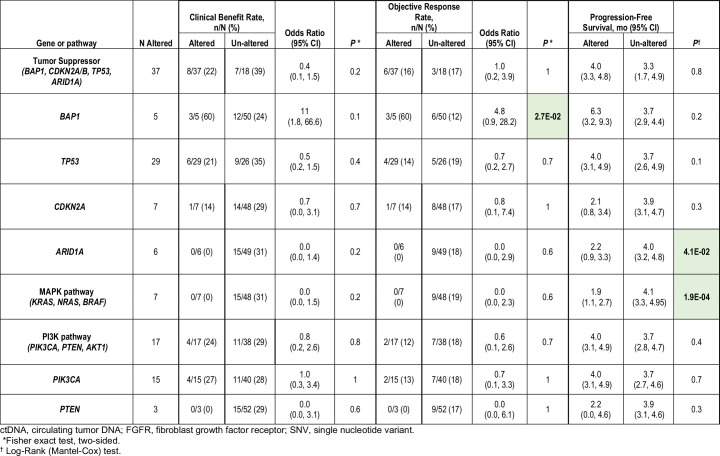
Baseline Co-alterations of Genes Belonging to Select Pathways in Patients with *FGFR* Fusions/ Rearrangements (Cohort A) and *FGFR* Actionable SNVs (Cohort B) Associated With Response (ctDNA only; N = 55)

**Extended Data Table 5:**
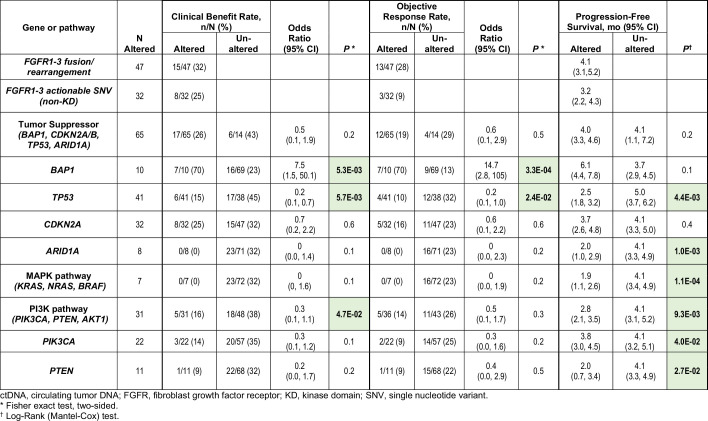
Baseline Co-alterations of Genes Belonging to Select Pathways in Patients with *FGFR* Fusions/ Rearrangements (Cohort A) and *FGFR* Actionable SNVs (Cohort B) Associated With Response (Combined Tissue and ctDNA; N = 79)

**Extended Data Table 6:**
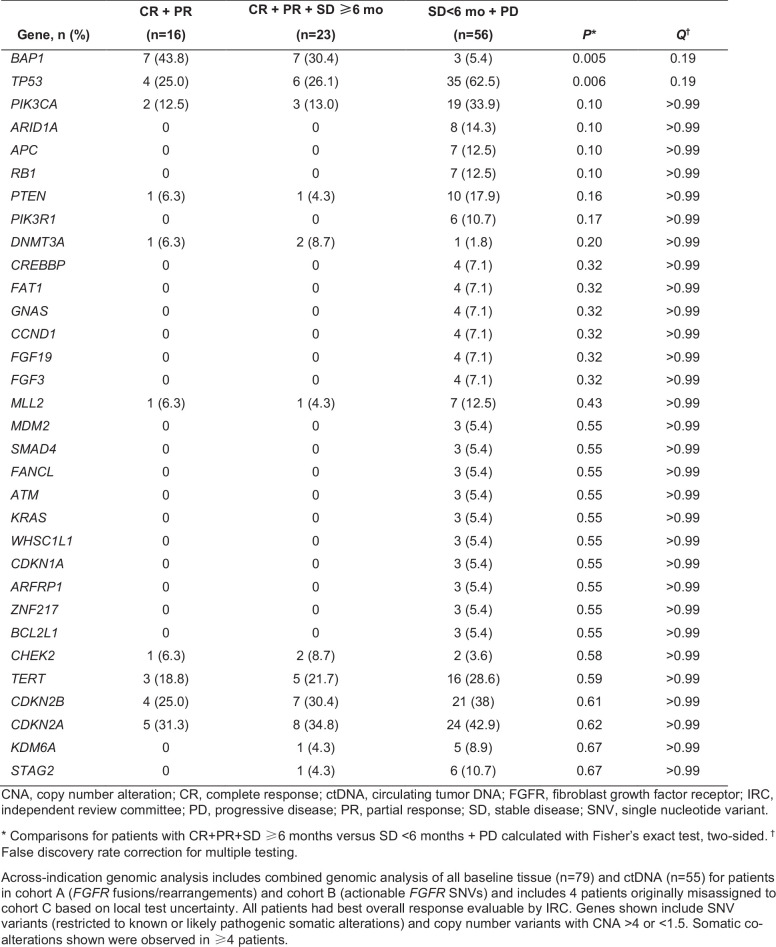
Baseline Co-alterations Associated With Response

**Extended Data Table 7:**
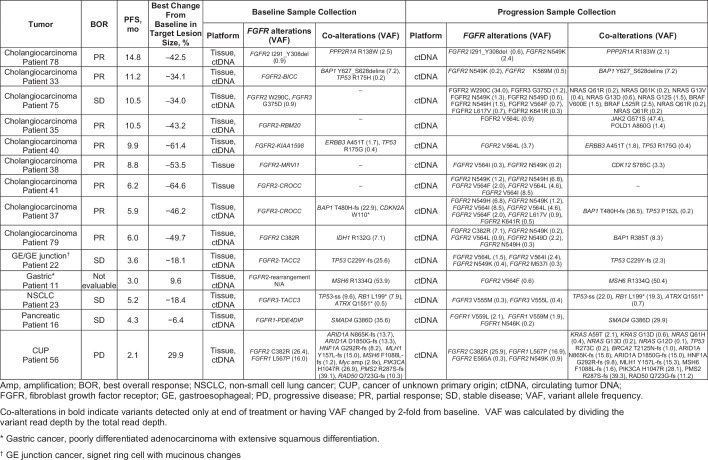
Acquired Resistance Mutations in FGFRs

## Data Availability

Incyte Corporation is committed to data sharing that advances science and medicine while protecting patient privacy. The study protocol with confidential information redacted is provided in the Supplementary Information. Qualified external scientific researchers may request anonymized datasets owned by Incyte for the purpose of conducting legitimate scientific research. Researchers may request anonymized datasets from any interventional study (except phase 1 studies) for which the product and indication have been approved on or after 1 January 2020 in at least one major market (for example, United States, EU and Japan). Data will be available for request after the primary publication or 2 years after the study has ended. Information on Incyte’s clinical trial data-sharing policy and instructions for submitting clinical trial data requests are available at https://www.incyte.com/Portals/0/Assets/Compliance%20and%20Transparency/clinical-trial-data-sharing.pdf?ver=2020-05-21-132838-960. Anonymized gene variant analyses are available through controlled access at dbGaP, accession number: phs003590.v1.p1.
