# Publisher Correction: Pemigatinib in previously treated solid tumors with activating *FGFR1–FGFR3* alterations: phase 2 FIGHT-207 basket trial

**DOI:** 10.1038/s41591-024-03072-w

**Published:** 2024-05-24

**Authors:** Jordi Rodón, Silvia Damian, Muhammad Furqan, Jesús García-Donas, Hiroo Imai, Antoine Italiano, Iben Spanggaard, Makoto Ueno, Tomoya Yokota, Maria Luisa Veronese, Natalia Oliveira, Xin Li, Aidan Gilmartin, Michael Schaffer, Lipika Goyal

**Affiliations:** 1 The University of Texas MD Anderson Cancer Center, Houston, TX USA; 2 Fondazione IRCCS Istituto Nazionale dei Tumori, Milan, Italy; 3 University of Iowa, Iowa City, IA USA; 4 Centro Integral Oncologico Clara Campal, Madrid, Spain; 5 Tohoku University Hospital, Sendai-Shi, Japan; 6 Institut Bergonié, Bordeaux, France; 7 Faculty of Medicine, University of Bordeaux, Bordeaux, France; 8 Rigshospitalet Copenhagen University Hospital, Copenhagen, Denmark; 9 Kanagawa Cancer Center, Yokohama, Japan; 10 Shizuoka Cancer Center, Shizuoka, Japan; 11 Incyte International Biosciences Sàrl, Morges, Switzerland; 12 Incyte Corporation, Wilmington, DE USA; 13 Mass General Cancer Center, Harvard Medical School, Boston, MA USA; 14 Stanford Cancer Center, Stanford School of Medicine, Stanford, CA USA

**Keywords:** Targeted therapies, Cancer genetics, Bladder cancer, Bile duct cancer, CNS cancer

Correction to: *Nature Medicine* 10.1038/s41591-024-02934-7, published online 6 May 2024

In the version of the article initially published, patient numbers and ratios of the trial cohorts were missing from the abstract and have now been added in the HTML and PDF versions of the article.

