# Supplementary material for: Pemigatinib in previously treated solid tumors with activating FGFR1–FGFR3 alterations: phase 2 FIGHT-207 basket trial
Source: Nat Med. 2024 May 6;30(6):1645–54. doi: 10.1038/s41591-024-02934-7 (PMC11186762; doi:10.1038/s41591-024-02934-7)

nature medicine

Supplementary information https://doi.org/10.1038/s41591-024-02934-7

Pemigatinib in previously treated solid tumors with activating *FGFR1–FGFR3* alterations: phase 2 FIGHT-207 basket trial

In the format provided by the authors and unedited

Supplementary Table 1. Demographics and Baseline Clinical Characteristics of Patients With FGFR Alteration Not Centrally Confirmed

	**Patients With *FGFR* Alteration Not Centrally Confirmed (n=4)**
Age, median (range), y	44.0 (36–68)
Women, n (%)	1 (25.0)
Race, n (%)	
White	0
Black/African American	0
Asian	4 (100.0)
ECOG PS, n (%)	
0	1 (25.0)
1	3 (75.0)
2	0
Current stage, n (%)	
Locally advanced	0
Metastatic	4 (100.0)
Prior radiation, n (%)	1 (25.0)
Prior surgery for cancer, n (%)	0
Local regional therapy, n (%)	0
Prior systemic therapy, n (%)	4 (100.0)
1	2 (50.0)
2	0

≥3	2 (50.0)
Solid tumor type, n (%)	
Cervical	1 (25.0)
Cholangiocarcinoma	1 (25.0)
Gallbladder	1 (25.0)
Thymic	1 (25.0)

ECOG PS, Eastern Cooperative Oncology Group performance status; FGFR, fibroblast growth factor receptor; FISH, fluorescence in situ hybridization; NGS, next-generation sequencing.

FGFR alterations were detected by liquid NGS analysis or FISH assays with breakapart *FGFR* probes but could not be confirmed with central tissue NGS.

Supplementary Table 2. Characteristics of Patients With Clinical Benefit*

Patient	Cohort	Tumor Type	*FGFR* Alteration†	BOR	Best % Change From BL in Sum of Target Lesion Sizes	PFS, mo
**Central nervous system tumors**						
44	A	Glioblastoma	*FGFR3-TACC3*	CR	−100.0	20.2‡
45	A	Glioblastoma	*FGFR3-TACC3*	PR	−100.0	8.4‡
100	C	Diffuse astrocytoma	*FGFR1* K656E	PR	−80.7	8.0
N/A§	C	Low-grade pediatric type glioma	*FGFR1* N546K	SD	N/A	6.2‡
**Gynecologic cancer**						
46	A	Cervical cancer	*FGFR3-TACC3*	PR	−100.0	6.4
30	A	Cervical cancer	*FGFR3-TACC3*	PR	−32.1	6.3
**Pancreatic cancer**						
43	A	Pancreatic adenocarcinoma	*FGFR2-PPHLN1*	PR	−72.1	8.4‡
39	A	Pancreatic adenocarcinoma	*FGFR2-KIAA1217* *FGFR2-TBC1D5*	PR	−54.9	8.9‡
32	A	Pancreatic adenocarcinoma	*FGFR2-KIAA1595*	PR	−33.3	6.3‡
**Cholangiocarcinoma**						
41	A	Cholangiocarcinoma	*FGFR2-CROCC*	PR	−64.6	6.2

Patient	Cohort	Tumor Type	*FGFR* Alteration†	BOR	Best % Change From BL in Sum of Target Lesion Sizes	PFS, mo
40	A	Cholangiocarcinoma	*FGFR2-KIAA1598*	PR	−61.4	9.9
38	A	Cholangiocarcinoma	*FGFR2-MRVI1*	PR	−53.5	8.8
79	B	Cholangiocarcinoma	*FGFR2* C382R	PR	−49.7	6.0
37	A	Cholangiocarcinoma	*FGFR2-BICC1*	PR	−46.2	5.9‡
35	A	Cholangiocarcinoma	*FGFR2-RBM20*	PR	−43.2	10.5
78	B	Cholangiocarcinoma	*FGFR2* I291, Y308 deletion	PR	−42.5	14.8
33	A	Cholangiocarcinoma	*FGFR2-BICC1*	PR	−34.1	11.2‡
75	B	Cholangiocarcinoma	*FGFR2* W290C	SD	−34.0	10.5
**Urothelial tract/bladder cancer**						
80	B	Urothelial tract/bladder cancer	*FGFR3* Y373C	PR	−50.8	6.2‡
68	B	Urothelial tract/bladder cancer	*FGFR3* G370C	SD	−10.2	6.3‡
64	B	Urothelial tract/bladder cancer	*FGFR3* G370C	SD	0	6.2‡
**Breast cancer**						
99	C	Breast cancer	*FGFR2* Q774*	SD	−25.0	18.7
97	C	Breast cancer	*FGFR1* I387V, *FGFR3* R349C	SD	−3.4	8.3‡
**Other cancers**						

Patient	Cohort	Tumor Type	*FGFR* Alteration†	BOR	Best % Change From BL in Sum of Target Lesion Sizes	PFS, mo
69	B	Submandibular gland cancer	*FGFR3* S371C	SD	−12.5	8.3
17	A	Head and neck cancer	*FGFR2-RBFOX2*	SD	−7.7	8.1

BL, baseline; BOR, best overall response; CR, complete response; FGFR, fibroblast growth factor receptor; PFS, progression-free survival; PR, partial response; SD, stable disease.
* Defined as objective response of any duration or stable disease ≥6 months.
† Includes *FGFR* alterations confirmed through both local and central genetic testing reports.
‡ Censored.
§ Postbaseline scan not available; therefore, the patient is not shown on the waterfall plot.

Supplementary Table 3. All treatment-emergent adverse events occurring in the safety population (N=111)

	Total (N=111)				
**Patients,* n (%)**	**All Grades**	**Grades 1 and 2**	**Grade 3**	**Grade 4**	**Grade 5**
Laboratory values and investigations					
Blood creatinine increased	18 (16.2)	17 (15.3)	1 (0.9)	0	0
Weight decreased	16 (14.4)	16 (14.4)	0	0	0
Alanine aminotransferase increased	14 (12.6)	12 (10.8)	2 (1.8)	0	0
Aspartate aminotransferase increased	14 (12.6)	9 (8.1)	5 (4.5)	0	0
Lipase increased	5 (4.5)	4 (3.6)	1 (0.9)	0	0
Blood alkaline phosphatase increased	4 (3.6)	4 (3.6)	0	0	0
Blood bilirubin increased	4 (3.6)	2 (1.8)	2 (1.8)	0	0
Lymphocyte count decreased	3 (2.7)	1 (0.9)	2 (1.8)	0	0
Neutrophil count decreased	3 (2.7)	2 (1.8)	0	1 (0.9)	0
Gamma-glutamyltransferase increased	2 (1.8)	2 (1.8)	0	0	0
Platelet count decreased	2 (1.8)	2 (1.8)	0	0	0
White blood cell count decreased	2 (1.8)	2 (1.8)	0	0	0
Amylase increased	1 (0.9)	1 (0.9)	0	0	0
Bilirubin conjugated increased	1 (0.9)	0	1 (0.9)	0	0
Blood chloride decreased	1 (0.9)	1 (0.9)	0	0	0
Blood creatine phosphokinase increased	1 (0.9)	0	1 (0.9)	0	0

Patients,* n (%)	Total (N=111)				
	All Grades	Grades 1 and 2	Grade 3	Grade 4	Grade 5
Blood magnesium decreased	1 (0.9)	1 (0.9)	0	0	0
Blood phosphorus increased	1 (0.9)	1 (0.9)	0	0	0
C-reactive protein increased	1 (0.9)	1 (0.9)	0	0	0
Electrocardiogram QT prolonged	1 (0.9)	1 (0.9)	0	0	0
Hemoglobin decreased	1 (0.9)	1 (0.9)	0	0	0
Hepatic enzyme increased	1 (0.9)	0	1 (0.9)	0	0
International normalized ratio increased	1 (0.9)	1 (0.9)	0	0	0
Neutrophil count increased	1 (0.9)	1 (0.9)	0	0	0
Urine leukocyte esterase positive	1 (0.9)	1 (0.9)	0	0	0
Vitamin D increased	1 (0.9)	1 (0.9)	0	0	0
Other TEAEs					
Hyperphosphatemia	93 (83.8)	92 (82.9)	1 (0.9)	0	0
Stomatitis	59 (53.2)	49 (44.1)	10 (9.0)	0	0
Alopecia	45 (40.5)	44 (39.6)	1 (0.9)	0	0
Diarrhea	43 (38.7)	42 (37.8)	1 (0.9)	0	0
Constipation	37 (33.3)	36 (32.4)	1 (0.9)	0	0
Dry mouth	32 (28.8)	32 (28.8)	0	0	0
Dysgeusia	30 (27.0)	30 (27.0)	0	0	0
Decreased appetite	28 (25.2)	23 (20.7)	5 (4.5)	0	0
Nausea	28 (25.2)	26 (23.4)	2 (1.8)	0	0
Asthenia	27 (24.3)	22 (19.8)	5 (4.5)	0	0

Patients,* n (%)	Total (N=111)				
	All Grades	Grades 1 and 2	Grade 3	Grade 4	Grade 5
Palmar-plantar erythrodysesthesia syndrome	26 (23.4)	20 (18.0)	6 (5.4)	0	0
Dry eye	25 (22.5)	22 (19.8)	3 (2.7)	0	0
Fatigue	24 (21.6)	19 (17.1)	5 (4.5)	0	0
Arthralgia	23 (20.7)	20 (18.0)	3 (2.7)	0	0
Vomiting	22 (19.8)	20 (18.0)	2 (1.8)	0	0
Dry skin	18 (16.2)	17 (15.3)	1 (0.9)	0	0
Abdominal pain	17 (15.3)	14 (12.6)	3 (2.7)	0	0
Onychomadesis	17 (15.3)	15 (13.5)	2 (1.8)	0	0
Urinary tract infection	17 (15.3)	14 (12.6)	3 (2.7)	0	0
Anemia	14 (12.6)	13 (11.7)	1 (0.9)	0	0
Edema peripheral	14 (12.6)	12 (10.8)	2 (1.8)	0	0
Paronychia	14 (12.6)	11 (9.9)	3 (2.7)	0	0
Nail discoloration	13 (11.7)	12 (10.8)	1 (0.9)	0	0
Cough	11 (9.9)	11 (9.9)	0	0	0
Hypophosphatemia	10 (9.0)	6 (5.4)	4 (3.6)	0	0
Myalgia	10 (9.0)	8 (7.2)	2 (1.8)	0	0
Nail disorder	10 (9.0)	9 (8.1)	1 (0.9)	0	0
Onycholysis	10 (9.0)	9 (8.1)	1 (0.9)	0	0
Pain in extremity	10 (9.0)	10 (9.0)	0	0	0
Back pain	9 (8.1)	7 (6.3)	2 (1.8)	0	0
Dyspnea	9 (8.1)	9 (8.1)	0	0	0

Patients,* n (%)	Total (N=111)				
	**All Grades**	**Grades 1 and 2**	**Grade 3**	**Grade 4**	**Grade 5**
Epistaxis	9 (8.1)	9 (8.1)	0	0	0
Hypercalcemia	9 (8.1)	6 (5.4)	1 (0.9)	2 (1.8)	0
Serous retinal detachment	9 (8.1)	7 (6.3)	2 (1.8)	0	0
Headache	8 (7.2)	7 (6.3)	1 (0.9)	0	0
Pyrexia	8 (7.2)	8 (7.2)	0	0	0
Vision blurred	8 (7.2)	8 (7.2)	0	0	0
Abdominal pain upper	7 (6.3)	7 (6.3)	0	0	0
Conjunctivitis	7 (6.3)	7 (6.3)	0	0	0
Dizziness	7 (6.3)	7 (6.3)	0	0	0
Hypomagnesemia	7 (6.3)	6 (5.4)	1 (0.9)	0	0
Hyponatremia	7 (6.3)	4 (3.6)	3 (2.7)	0	0
Dyspepsia	6 (5.4)	6 (5.4)	0	0	0
Hypotension	6 (5.4)	5 (4.5)	1 (0.9)	0	0
Pruritus	6 (5.4)	6 (5.4)	0	0	0
Urinary retention	6 (5.4)	6 (5.4)	0	0	0
Dehydration	5 (4.5)	4 (3.6)	1 (0.9)	0	0
Malaise	5 (4.5)	5 (4.5)	0	0	0
Rash	5 (4.5)	5 (4.5)	0	0	0
Renal failure	5 (4.5)	4 (3.6)	1 (0.9)	0	0
Taste disorder	5 (4.5)	5 (4.5)	0	0	0
Cellulitis	4 (3.6)	2 (1.8)	1 (0.9)	1 (0.9)	0

Patients,* n (%)	Total (N=111)				
	All Grades	Grades 1 and 2	Grade 3	Grade 4	Grade 5
COVID-19	4 (3.6)	4 (3.6)	0	0	0
Cystitis	4 (3.6)	4 (3.6)	0	0	0
Dysuria	4 (3.6)	4 (3.6)	0	0	0
Erythema	4 (3.6)	4 (3.6)	0	0	0
Fall	4 (3.6)	4 (3.6)	0	0	0
General physical health deterioration	4 (3.6)	1 (0.9)	0	0	3 (2.7)
Hyperglycemia	4 (3.6)	4 (3.6)	0	0	0
Hypertension	4 (3.6)	3 (2.7)	1 (0.9)	0	0
Hypokalemia	4 (3.6)	2 (1.8)	2 (1.8)	0	0
Insomnia	4 (3.6)	4 (3.6)	0	0	0
Keratitis	4 (3.6)	3 (2.7)	1 (0.9)	0	0
Muscle spasms	4 (3.6)	4 (3.6)	0	0	0
Onychalgia	4 (3.6)	4 (3.6)	0	0	0
Oropharyngeal pain	4 (3.6)	4 (3.6)	0	0	0
Thrombocytopenia	4 (3.6)	4 (3.6)	0	0	0
Vitamin D deficiency	4 (3.6)	4 (3.6)	0	0	0
Acute kidney injury	3 (2.7)	2 (1.8)	1 (0.9)	0	0
Agitation	3 (2.7)	3 (2.7)	0	0	0
Ascites	3 (2.7)	2 (1.8)	1 (0.9)	0	0
Cheilitis	3 (2.7)	3 (2.7)	0	0	0
Confusional state	3 (2.7)	1 (0.9)	1 (0.9)	0	1 (0.9)

Patients,* n (%)	Total (N=111)				
	All Grades	Grades 1 and 2	Grade 3	Grade 4	Grade 5
Depression	3 (2.7)	3 (2.7)	0	0	0
Dysphagia	3 (2.7)	3 (2.7)	0	0	0
Edema	3 (2.7)	3 (2.7)	0	0	0
Gastroesophageal reflux disease	3 (2.7)	3 (2.7)	0	0	0
Hot flush	3 (2.7)	3 (2.7)	0	0	0
Hypertrichosis	3 (2.7)	3 (2.7)	0	0	0
Hyperuricemia	3 (2.7)	3 (2.7)	0	0	0
Hypocalcemia	3 (2.7)	3 (2.7)	0	0	0
Lymphopenia	3 (2.7)	1 (0.9)	1 (0.9)	1 (0.9)	0
Muscular weakness	3 (2.7)	3 (2.7)	0	0	0
Nail infection	3 (2.7)	3 (2.7)	0	0	0
Nasal dryness	3 (2.7)	3 (2.7)	0	0	0
Osteoarthritis	3 (2.7)	3 (2.7)	0	0	0
Paraesthesia	3 (2.7)	2 (1.8)	1 (0.9)	0	0
Pollakiuria	3 (2.7)	3 (2.7)	0	0	0
Presyncope	3 (2.7)	2 (1.8)	1 (0.9)	0	0
Punctate keratitis	3 (2.7)	2 (1.8)	1 (0.9)	0	0
Retinal detachment	3 (2.7)	2 (1.8)	1 (0.9)	0	0
Sciatica	3 (2.7)	3 (2.7)	0	0	0
Sepsis	3 (2.7)	1 (0.9)	1 (0.9)	0	1 (0.9)
Xerosis	3 (2.7)	3 (2.7)	0	0	0

Patients,* n (%)	Total (N=111)				
	All Grades	Grades 1 and 2	Grade 3	Grade 4	Grade 5
Abdominal distension	2 (1.8)	2 (1.8)	0	0	0
Abdominal pain lower	2 (1.8)	2 (1.8)	0	0	0
Anxiety	2 (1.8)	2 (1.8)	0	0	0
Aphthous ulcer	2 (1.8)	2 (1.8)	0	0	0
Arthritis	2 (1.8)	1 (0.9)	1 (0.9)	0	0
Balance disorder	2 (1.8)	1 (0.9)	1 (0.9)	0	0
Brain edema	2 (1.8)	2 (1.8)	0	0	0
Cancer pain	2 (1.8)	2 (1.8)	0	0	0
Cataract	2 (1.8)	2 (1.8)	0	0	0
Chronic kidney disease	2 (1.8)	0	2 (1.8)	0	0
Corneal erosion	2 (1.8)	2 (1.8)	0	0	0
Cutaneous calcification	2 (1.8)	1 (0.9)	1 (0.9)	0	0
Delirium	2 (1.8)	2 (1.8)	0	0	0
Dysphonia	2 (1.8)	2 (1.8)	0	0	0
Face edema	2 (1.8)	2 (1.8)	0	0	0
Hematemesis	2 (1.8)	1 (0.9)	1 (0.9)	0	0
Hematuria	2 (1.8)	2 (1.8)	0	0	0
Hepatic function abnormal	2 (1.8)	2 (1.8)	0	0	0
Hiccups	2 (1.8)	2 (1.8)	0	0	0
Hypoesthesia	2 (1.8)	2 (1.8)	0	0	0
Maculopathy	2 (1.8)	2 (1.8)	0	0	0

Patients,* n (%)	Total (N=111)				
	All Grades	Grades 1 and 2	Grade 3	Grade 4	Grade 5
Musculoskeletal chest pain	2 (1.8)	2 (1.8)	0	0	0
Nail dystrophy	2 (1.8)	2 (1.8)	0	0	0
Nail toxicity	2 (1.8)	2 (1.8)	0	0	0
Nasal congestion	2 (1.8)	2 (1.8)	0	0	0
Odynophagia	2 (1.8)	2 (1.8)	0	0	0
Onychoclasis	2 (1.8)	1 (0.9)	1 (0.9)	0	0
Oral candidiasis	2 (1.8)	2 (1.8)	0	0	0
Oral herpes	2 (1.8)	2 (1.8)	0	0	0
Pelvic pain	2 (1.8)	2 (1.8)	0	0	0
Peripheral sensory neuropathy	2 (1.8)	2 (1.8)	0	0	0
Pharyngitis	2 (1.8)	2 (1.8)	0	0	0
Pneumothorax	2 (1.8)	1 (0.9)	1 (0.9)	0	0
Pulmonary embolism	2 (1.8)	0	2 (1.8)	0	0
Rectal hemorrhage	2 (1.8)	2 (1.8)	0	0	0
Skin exfoliation	2 (1.8)	2 (1.8)	0	0	0
Somnolence	2 (1.8)	2 (1.8)	0	0	0
Subretinal fluid	2 (1.8)	2 (1.8)	0	0	0
Visual impairment	2 (1.8)	2 (1.8)	0	0	0
Xerophthalmia	2 (1.8)	2 (1.8)	0	0	0
Abdominal discomfort	1 (0.9)	1 (0.9)	0	0	0
Abdominal wall hematoma	1 (0.9)	1 (0.9)	0	0	0

Patients,* n (%)	Total (N=111)				
	All Grades	Grades 1 and 2	Grade 3	Grade 4	Grade 5
Abdominal wall infection	1 (0.9)	1 (0.9)	0	0	0
Acute respiratory failure	1 (0.9)	0	0	0	1 (0.9)
Age-related macular degeneration	1 (0.9)	1 (0.9)	0	0	0
Ageusia	1 (0.9)	1 (0.9)	0	0	0
Aphasia	1 (0.9)	1 (0.9)	0	0	0
Asthenopia	1 (0.9)	1 (0.9)	0	0	0
Ataxia	1 (0.9)	0	1 (0.9)	0	0
Atelectasis	1 (0.9)	1 (0.9)	0	0	0
Bacteremia	1 (0.9)	1 (0.9)	0	0	0
Biliary obstruction	1 (0.9)	0	1 (0.9)	0	0
Blepharitis	1 (0.9)	1 (0.9)	0	0	0
Bradycardia	1 (0.9)	1 (0.9)	0	0	0
Bursitis	1 (0.9)	1 (0.9)	0	0	0
Cartilage injury	1 (0.9)	1 (0.9)	0	0	0
Cell death	1 (0.9)	1 (0.9)	0	0	0
Chest discomfort	1 (0.9)	1 (0.9)	0	0	0
Chills	1 (0.9)	1 (0.9)	0	0	0
Cholangitis	1 (0.9)	0	1 (0.9)	0	0
Cholestasis	1 (0.9)	0	1 (0.9)	0	0
Cognitive disorder	1 (0.9)	1 (0.9)	0	0	0
Conjunctival edema	1 (0.9)	1 (0.9)	0	0	0

Patients,* n (%)	Total (N=111)				
	All Grades	Grades 1 and 2	Grade 3	Grade 4	Grade 5
Contusion	1 (0.9)	1 (0.9)	0	0	0
Corneal disorder	1 (0.9)	1 (0.9)	0	0	0
Corneal infection	1 (0.9)	1 (0.9)	0	0	0
Cystitis noninfective	1 (0.9)	1 (0.9)	0	0	0
Dermatitis	1 (0.9)	1 (0.9)	0	0	0
Diplopia	1 (0.9)	1 (0.9)	0	0	0
Dry throat	1 (0.9)	1 (0.9)	0	0	0
Early satiety	1 (0.9)	1 (0.9)	0	0	0
Epilepsy	1 (0.9)	0	1 (0.9)	0	0
Erectile dysfunction	1 (0.9)	1 (0.9)	0	0	0
Esophagitis	1 (0.9)	1 (0.9)	0	0	0
Eye discharge	1 (0.9)	1 (0.9)	0	0	0
Eye disorder	1 (0.9)	1 (0.9)	0	0	0
Eye hemorrhage	1 (0.9)	1 (0.9)	0	0	0
Eye infection	1 (0.9)	1 (0.9)	0	0	0
Eye irritation	1 (0.9)	1 (0.9)	0	0	0
Eyelid ptosis	1 (0.9)	1 (0.9)	0	0	0
Fascial infection	1 (0.9)	0	1 (0.9)	0	0
Febrile neutropenia	1 (0.9)	0	1 (0.9)	0	0
Flank pain	1 (0.9)	1 (0.9)	0	0	0
Foot fracture	1 (0.9)	1 (0.9)	0	0	0

Patients,* n (%)	Total (N=111)				
	All Grades	Grades 1 and 2	Grade 3	Grade 4	Grade 5
Fungal skin infection	1 (0.9)	1 (0.9)	0	0	0
Gastritis	1 (0.9)	1 (0.9)	0	0	0
Gastrointestinal pain	1 (0.9)	1 (0.9)	0	0	0
Gastrointestinal stoma complication	1 (0.9)	1 (0.9)	0	0	0
Gastrostomy failure	1 (0.9)	1 (0.9)	0	0	0
Genital dysesthesia	1 (0.9)	1 (0.9)	0	0	0
Glossitis	1 (0.9)	1 (0.9)	0	0	0
Granulomatous dermatitis	1 (0.9)	1 (0.9)	0	0	0
Growth of eyelashes	1 (0.9)	1 (0.9)	0	0	0
Hemoptysis	1 (0.9)	1 (0.9)	0	0	0
Herpes simplex reactivation	1 (0.9)	1 (0.9)	0	0	0
Hydronephrosis	1 (0.9)	0	1 (0.9)	0	0
Hyperammonemia	1 (0.9)	1 (0.9)	0	0	0
Hyperbilirubinemia	1 (0.9)	1 (0.9)	0	0	0
Hyperkalemia	1 (0.9)	1 (0.9)	0	0	0
Hyperparathyroidism	1 (0.9)	1 (0.9)	0	0	0
Hypertransaminasemia	1 (0.9)	0	1 (0.9)	0	0
Hypoalbuminemia	1 (0.9)	1 (0.9)	0	0	0
Hypoglycemia	1 (0.9)	1 (0.9)	0	0	0
Hypothyroidism	1 (0.9)	1 (0.9)	0	0	0
Hypoxia	1 (0.9)	1 (0.9)	0	0	0

Patients,* n (%)	Total (N=111)				
	All Grades	Grades 1 and 2	Grade 3	Grade 4	Grade 5
Implant site infection	1 (0.9)	0	1 (0.9)	0	0
Inadequate analgesia	1 (0.9)	0	1 (0.9)	0	0
Infection	1 (0.9)	1 (0.9)	0	0	0
Infusion site laceration	1 (0.9)	1 (0.9)	0	0	0
Infusion site reaction	1 (0.9)	1 (0.9)	0	0	0
Intermenstrual bleeding	1 (0.9)	1 (0.9)	0	0	0
Intestinal obstruction	1 (0.9)	0	1 (0.9)	0	0
Iron deficiency anemia	1 (0.9)	1 (0.9)	0	0	0
Jaundice	1 (0.9)	0	1 (0.9)	0	0
Joint stiffness	1 (0.9)	1 (0.9)	0	0	0
Keratopathy	1 (0.9)	1 (0.9)	0	0	0
Lacrimal disorder	1 (0.9)	1 (0.9)	0	0	0
Lacrimation increased	1 (0.9)	1 (0.9)	0	0	0
Laryngeal inflammation	1 (0.9)	1 (0.9)	0	0	0
Lip infection	1 (0.9)	1 (0.9)	0	0	0
Liver disorder	1 (0.9)	0	1 (0.9)	0	0
Localized edema	1 (0.9)	1 (0.9)	0	0	0
Long QT syndrome	1 (0.9)	0	1 (0.9)	0	0
Macular edema	1 (0.9)	1 (0.9)	0	0	0
Macular pseudohole	1 (0.9)	1 (0.9)	0	0	0
Mastitis	1 (0.9)	1 (0.9)	0	0	0

Patients,* n (%)	Total (N=111)				
	**All Grades**	**Grades 1 and 2**	**Grade 3**	**Grade 4**	**Grade 5**
Medical device site pain	1 (0.9)	1 (0.9)	0	0	0
Memory impairment	1 (0.9)	1 (0.9)	0	0	0
Metastases to central nervous system	1 (0.9)	0	1 (0.9)	0	0
Microcytic anemia	1 (0.9)	0	1 (0.9)	0	0
Mucosal inflammation	1 (0.9)	1 (0.9)	0	0	0
Musculoskeletal pain	1 (0.9)	1 (0.9)	0	0	0
Nail ridging	1 (0.9)	1 (0.9)	0	0	0
Nasopharyngitis	1 (0.9)	1 (0.9)	0	0	0
Neck pain	1 (0.9)	1 (0.9)	0	0	0
Nephrolithiasis	1 (0.9)	1 (0.9)	0	0	0
Neuropathy peripheral	1 (0.9)	1 (0.9)	0	0	0
Neutropenia	1 (0.9)	0	1 (0.9)	0	0
Obstruction gastric	1 (0.9)	0	1 (0.9)	0	0
Ocular toxicity	1 (0.9)	1 (0.9)	0	0	0
Oral discomfort	1 (0.9)	1 (0.9)	0	0	0
Oral fungal infection	1 (0.9)	1 (0.9)	0	0	0
Otitis externa	1 (0.9)	1 (0.9)	0	0	0
Otorrhea	1 (0.9)	1 (0.9)	0	0	0
Palpitations	1 (0.9)	1 (0.9)	0	0	0
Pancreatic failure	1 (0.9)	1 (0.9)	0	0	0
Pancreatitis	1 (0.9)	0	1 (0.9)	0	0

Patients,* n (%)	Total (N=111)				
	All Grades	Grades 1 and 2	Grade 3	Grade 4	Grade 5
Parotitis	1 (0.9)	0	1 (0.9)	0	0
Pathological fracture	1 (0.9)	0	1 (0.9)	0	0
Penile pain	1 (0.9)	1 (0.9)	0	0	0
Perineal pain	1 (0.9)	1 (0.9)	0	0	0
Peripheral artery occlusion	1 (0.9)	1 (0.9)	0	0	0
Peripheral ischemia	1 (0.9)	0	1 (0.9)	0	0
Peripheral swelling	1 (0.9)	1 (0.9)	0	0	0
Photophobia	1 (0.9)	1 (0.9)	0	0	0
Photopsia	1 (0.9)	1 (0.9)	0	0	0
Pigmentation disorder	1 (0.9)	1 (0.9)	0	0	0
Pneumonia	1 (0.9)	0	1 (0.9)	0	0
Post procedural hematuria	1 (0.9)	0	1 (0.9)	0	0
Procedural pain	1 (0.9)	1 (0.9)	0	0	0
Pseudomonas infection	1 (0.9)	1 (0.9)	0	0	0
Psychomotor skills impaired	1 (0.9)	1 (0.9)	0	0	0
Pyelonephritis acute	1 (0.9)	0	1 (0.9)	0	0
Rash erythematous	1 (0.9)	1 (0.9)	0	0	0
Rash macular	1 (0.9)	1 (0.9)	0	0	0
Renal colic	1 (0.9)	1 (0.9)	0	0	0
Renal impairment	1 (0.9)	1 (0.9)	0	0	0
Retinal cyst	1 (0.9)	1 (0.9)	0	0	0

Patients,* n (%)	Total (N=111)				
	All Grades	Grades 1 and 2	Grade 3	Grade 4	Grade 5
Retinal hemorrhage	1 (0.9)	1 (0.9)	0	0	0
Retinopathy	1 (0.9)	1 (0.9)	0	0	0
Rhabdomyolysis	1 (0.9)	0	1 (0.9)	0	0
Rhinitis	1 (0.9)	1 (0.9)	0	0	0
Rhinorrhea	1 (0.9)	1 (0.9)	0	0	0
Seizure	1 (0.9)	1 (0.9)	0	0	0
Sinus pain	1 (0.9)	1 (0.9)	0	0	0
Sinus tachycardia	1 (0.9)	1 (0.9)	0	0	0
Sinusitis	1 (0.9)	0	1 (0.9)	0	0
Skin abrasion	1 (0.9)	1 (0.9)	0	0	0
Skin infection	1 (0.9)	1 (0.9)	0	0	0
Skin irritation	1 (0.9)	1 (0.9)	0	0	0
Skin ulcer	1 (0.9)	0	1 (0.9)	0	0
Sleep disorder	1 (0.9)	1 (0.9)	0	0	0
Speech disorder	1 (0.9)	1 (0.9)	0	0	0
Spinal cord compression	1 (0.9)	0	1 (0.9)	0	0
Stent malfunction	1 (0.9)	0	1 (0.9)	0	0
Stevens-Johnson syndrome	1 (0.9)	0	1 (0.9)	0	0
Swollen tongue	1 (0.9)	1 (0.9)	0	0	0
Syncope	1 (0.9)	0	1 (0.9)	0	0
Systemic inflammatory response syndrome	1 (0.9)	1 (0.9)	0	0	0

Patients,* n (%)	Total (N=111)				
	**All Grades**	**Grades 1 and 2**	**Grade 3**	**Grade 4**	**Grade 5**
Tinnitus	1 (0.9)	1 (0.9)	0	0	0
Tongue discomfort	1 (0.9)	1 (0.9)	0	0	0
Tonsillitis	1 (0.9)	1 (0.9)	0	0	0
Toothache	1 (0.9)	1 (0.9)	0	0	0
Tremor	1 (0.9)	1 (0.9)	0	0	0
Trichiasis	1 (0.9)	1 (0.9)	0	0	0
Ulcerative keratitis	1 (0.9)	0	1 (0.9)	0	0
Upper respiratory tract infection	1 (0.9)	1 (0.9)	0	0	0
Ureteric obstruction	1 (0.9)	0	1 (0.9)	0	0
Urinary hesitation	1 (0.9)	1 (0.9)	0	0	0
Urinary incontinence	1 (0.9)	1 (0.9)	0	0	0
Urinary tract obstruction	1 (0.9)	0	1 (0.9)	0	0
Vaccination site pain	1 (0.9)	1 (0.9)	0	0	0
Vascular access site pain	1 (0.9)	1 (0.9)	0	0	0
Vertigo	1 (0.9)	1 (0.9)	0	0	0
Visual acuity reduced	1 (0.9)	1 (0.9)	0	0	0
Vulval ulceration	1 (0.9)	1 (0.9)	0	0	0
Vulvitis	1 (0.9)	1 (0.9)	0	0	0
Vulvovaginal candidiasis	1 (0.9)	1 (0.9)	0	0	0
Xeroderma	1 (0.9)	1 (0.9)	0	0	0

MedDRA, Medical Dictionary for Regulatory Activities; TEAE, treatment-emergent adverse event.
* Patients were counted once under each MedDRA preferred term.

Supplementary Table 4. Characteristics of Patients With Concurrent *FGFR* Amplifications

Patient	Cohort	Histology	Enrolled *FGFR* Alteration	Amplified *FGFR* Gene*	Copy Number	BOR	PFS, mo
12	A	Endometrial cancer	*BAG4-FGFR1*	*FGFR1*	12	SD	4.1
84	C	Cholangiocarcinoma	*FGFR3* K659R	*FGFR3*	12	PD	2.1
86	C	Breast cancer	*FGFR1* N546K	*FGFR1*	8	PD	1.4
98	C	Gastroesophageal/ gastroesophageal junction cancer	*FGFR2* E769fs	*FGFR2*	48	SD	6.2
97	C	Breast cancer	*FGFR1* I387V; *FGFR3* R439C	*FGFR1*	18	SD	8.3†
3	A	Esophageal cancer	*FGFR1-WHSC1L1*	*FGFR2*	15	PD	1.6
23	A	Non-small cell lung cancer	*FGFR3-TACC3*	*FGFR1*	8	SD	5.2
21	A	Cancer of unknown primary	*FGFR2* rearrangement	*FGFR3*	9	SD	3.6
49	B	Uterine cancer	*FGFR2* V395D	*FGFR1*	10	PD	1.2

BOR, best overall response; FGFR, fibroblast growth factor receptor; PD, progressive disease; PFS, progression-free survival; SD, stable disease.
* *FGFR* amplification defined as copy number ≥6
† Censored.

Supplementary Table 5. Pooled Analysis of Baseline Co-Alterations Associated with Response Across Histologies in Tumor Tissue From Patients in FIGHT-207 and FIGHT-101

Gene	N Altered	Objective Response, n/N (%)		Odds Ratio (95% CI)	*P**	*Q*†
		Altered	Unaltered			
BAP1	11	8/11 (73)	18/81 (16)	13.4 (2.8, 88.6)	**<0.001**	0.017
TP53	27	0/27 (0)	21/65 (32)	0 (0, 0.4)	**<0.001**	0.017
APC	7	0/7 (0)	21/85 (25)	0 (0, 2.3)	0.34	>0.99
RB1	7	0/7 (0)	21/85 (25)	0 (0, 2.3)	0.34	>0.99
PIK3CA	16	2/16 (13)	19/76 (25)	0.4 (0.04, 2.2)	0.35	>0.99
MCL1	3	1/3 (33)	20/89 (23)	1.7 (0.03, 34.5)	0.54	>0.99
EGFR	3	1/3 (33)	20/89 (23)	1.7 (0.03, 34.5)	0.54	>0.99
PPP2R1A	3	1/3 (33)	20/89 (23)	1.7 (0.03, 34.5)	0.54	>0.99
RBM10	3	1/3 (33)	20/89 (23)	1.7 (0.03, 34.5)	0.54	>0.99
FGF4	3	1/3 (33)	20/89 (23)	1.7 (0.03, 34.5)	0.54	>0.99
MTAP	20	3/20 (15)	18/72 (25)	0.5 (0.09, 2.2)	0.55	>0.99
MDM2	4	0/4 (0)	21/88 (24)	0 (0, 5.2)	0.57	>0.99
SMAD4	4	0/4 (0)	21/88 (24)	0 (0, 5.2)	0.57	>0.99
CREBBP	4	0/4 (0)	21/88 (24)	0 (0, 5.2)	0.57	>0.99
PIK3R1	4	0/4 (0)	21/88 (24)	0 (0, 5.2)	0.57	>0.99
CDKN1A	4	0/4 (0)	21/88 (24)	0 (0, 5.2)	0.57	>0.99
TSC1	5	0/5 (0)	21/87 (24)	0 (0, 3.7)	0.59	>0.99
ARID1A	8	1/8 (13)	20/84 (24)	0.5 (0.01, 4.0)	0.68	>0.99

PTEN	9	1/9 (11)	20/83 (24)	0.4 (0.01, 3.3)	0.68	>0.99
MLL2	10	3/10 (30)	18/82 (22)	1.5 (0.2, 7.5)	0.69	>0.99
KDM6A	11	3/11 (27)	18/81 (22)	1.3 (0.2, 6.2)	0.71	>0.99
TERT	27	7/27 (26)	14/65 (22)	1.3 (0.4, 4.0)	0.79	>0.99
DNMT3A	4	1/4 (25)	20/88 (23)	1.1 (0.02, 15.0)	>0.99	>0.99
STAG2	9	2/9 (22)	19/83 (23)	1.0 (0.09, 5.7)	>0.99	>0.99
ATM	3	0/3 (0)	21/89 (24)	0 (0, 8.3)	>0.99	>0.99
MYC	6	1/6 (17)	20/86 (23)	0.7 (0.01, 6.4)	>0.99	>0.99
ARFRP1	3	0/3 (0)	21/89 (24)	0 (0, 8.3)	>0.99	>0.99
ZNF217	3	0/3 (0)	21/89 (24)	0 (0, 8.3)	>0.99	>0.99
BCL2L1	3	0/3 (0)	21/89 (24)	0 (0, 8.3)	>0.99	>0.99
FGF19	5	1/5 (20)	20/87 (23)	0.8 (0.02, 9.1)	>0.99	>0.99
FGF3	5	1/5 (20)	20/87 (23)	0.8 (0.02, 9.1)	>0.99	>0.99

Pooled dataset includes evaluable patients from the current FIGHT-207 study (n=72) and the open-label, phase 1/2, first-in-human FIGHT-101 study (n=20) irrespective of histology with *FGFR* fusions/rearrangements or mutations, pemigatinib ≥13.5 mg treatment, and best overall responses reported

* Fisher’s exact test,two-sided, for count data.

† False discovery rate correction for multiple testing.

Supplementary Table 6. Pooled Analysis of Baseline Co-Alterations Associated with Response in Tumor Tissue From Patients With Cholangiocarcinoma in FIGHT-207 and FIGHT-202

Gene	N Altered	Objective Response, n/N (%)		Odds Ratio (95% CI)	*P**	*Q*†
		Altered	Unaltered			
TP53	9	0/9 (0)	46/106 (43)	0 (0, 0.7)	**0.011**	0.91
CDKN2B	17	3/17 (18)	43/98 (44)	0.3 (0.05, 1.1)	0.059	>0.99
BAP1	44	22/44 (50)	24/71 (34)	2.0 (0.9, 4.5)	0.12	>0.99
CDKN2A	23	6/23 (26)	40/92 (44)	0.5 (0.1, 1.4)	0.16	>0.99
TERT	3	0/3 (0)	46/112 (41)	0 (0, 3.6)	0.3	>0.99
LYN	3	2/3 (67)	44/112 (39)	3.1 (0.2, 185)	0.6	>0.99
MTAP	4	1/4 (25)	45/111 (41)	0.5 (0.01, 6.4)	0.7	>0.99
IDH1	7	2/7 (29)	44/108 (41)	0.6 (0.05, 3.8)	0.7	>0.99
MCL1	8	4/8 (50)	42/107 (39)	1.5 (0.3, 8.8)	0.71	>0.99
PIK3CA	10	3/10 (30)	43/105 (41)	0.6 (0.1, 2.9)	0.74	>0.99
PBRM1	10	3/10 (30)	43/105 (41)	0.6 (0.1, 2.9)	0.74	>0.99
MYC	9	3/9 (33)	43/106 (41)	0.7 (0.1, 3.7)	0.74	>0.99
PTEN	5	2/5 (40)	44/110 (40)	1.0 (0.08, 9.1)	>0.99	>0.99
LRP1B	3	1/3 (33)	45/112 (40)	0.8 (0.01, 14.7)	>0.99	>0.99
FANCG	3	1/3 (33)	45/112 (40)	0.8 (0.01, 14.7)	>0.99	>0.99
FOXP1	3	1/3 (33)	45/112 (40)	0.8 (0.01, 14.7)	>0.99	>0.99

Pooled dataset includes evaluable patients from the current FIGHT-207 study (n=11) and the open-label, phase 2 FIGHT-202 study (n=104) with cholangiocarcinoma and best overall responses reported.
* Fisher's exact test, two-sided, for count data.
† False discovery rate correction for multiple testing.

Supplementary Table 7. Pooled Analysis of Baseline Co-Alterations Associated with Response in Tumor Tissue From Patients With Urothelial Carcinoma in FIGHT-207 and FIGHT-201

Gene	N Altered	Objective Response, n/N (%)		Odds Ratio (95% CI)	*P**	*Q*†
		Altered	Unaltered			
TSC1	36	3/36 (8)	33/126 (26)	0.3 (0.05, 0.9)	**0.023**	>0.99
CDKN1A	22	1/22 (5)	35/140 (25)	0.1 (0, 1.0)	**0.029**	>0.99
MLL2	37	12/37 (32)	24/125 (19)	2.0 (0.8, 4.9)	0.11	>0.99
EP300	17	1/17 (6.0)	35/145 (24)	0.2 (0, 1.4)	0.12	>0.99
ARID1A	34	11/34 (32)	25/128 (20)	2.0 (0.8, 4.9)	0.16	>0.99
NCOR1	7	3/7 (43)	33/155 (21)	2.8 (0.4, 17.1)	0.18	>0.99
CCND1	14	1/14 (7)	35/148 (24)	0.3 (0.01, 1.8)	0.20	>0.99
FGF19	14	1/14 (7)	35/148 (24)	0.3 (0.01, 1.8)	0.20	>0.99
SMARCA4	8	0/8 (0)	36/154 (23)	0 (0, 2.0)	0.20	>0.99
LRP1B	8	0/8 (0)	36/154 (23)	0 (0, 2.0)	0.20	>0.99
CDKN2B	89	23/89 (26)	13/73 (18)	1.6 (0.7, 3.8)	0.26	>0.99
STAG2	37	11/37 (30)	25/125 (20)	1.7 (0.7, 4.1)	0.26	>0.99

ATM	11	4/11 (36)	32/151 (21.2)	2.1 (0.4, 8.9)	0.26	>0.99
MLL3	23	7/23 (30)	29/139 (21)	1.7 (0.5, 4.8)	0.29	>0.99
FGF3	12	1/12 (8)	35/150 (23)	0.3 (0.01, 2.2)	0.30	>0.99
GNA13	5	2/5 (40)	34/157 (22)	2.4 (0.2, 21.8)	0.31	>0.99
PIK3CA	28	4/28 (14)	32/134 (24)	0.5 (0.1, 1.7)	0.33	>0.99
CDKN2A	100	25/100 (25)	11/62 (18)	1.5 (0.7, 3.8)	0.33	>0.99
ZNF703	7	0/7 (0)	36/155 (23)	0 (0, 2.4)	0.35	>0.99
CREBBP	16	5/16 (31)	31/146 (21)	1.7 (0.4, 5.7)	0.35	>0.99
DM6A	56	10/56 (18)	26/106 (25)	0.7 (0.3, 1.6)	0.43	>0.99
FGF4	11	1/11 (9)	35/151 (23)	0.3 (0.01, 2.5)	0.46	>0.99
PTEN	10	1/10 (10)	35/152 (23)	0.4 (0.01, 2.9)	0.46	>0.99
SF3B1	5	0/5 (0)	36/157 (23)	0 (0, 3.8)	0.59	>0.99
MDM2	25	4/25 (16)	32/137 (23)	0.6 (0.2, 2.1)	0.60	>0.99
FRS2	25	4/25 (16)	32/137 (23)	0.6 (0.2, 2.1)	0.60	>0.99
CHEK2	7	2/7 (29)	34/155 (22)	1.4 (0.1, 9.2)	0.65	>0.99

TP53	42	8/42 (19)	28/120 (23)	0.8 (0.3, 2.0)	0.67	>0.99
RB1	9	1/9 (11)	35/153 (23)	0.4 (0.01, 3.3)	0.69	>0.99
ERBB2	9	1/9 (11)	35/153 (23)	0.4 (0.01, 3.3)	0.69	>0.99
FAT1	8	1/8 (13)	35/154 (23)	0.5 (0.01, 4.0)	0.69	>0.99
MUTYH	6	1/6 (17)	35/156 (22)	0.7 (0.01, 6.5)	>0.99	>0.99
MTAP	7	1/7 (14)	35/155 (23)	0.6 (0.01, 5.0)	>0.99	>0.99
FBXW7	10	2/10 (20)	34/152 (22)	0.9 (0.09, 4.6)	>0.99	>0.99
BAP1	5	1/5 (20)	35/157 (22)	0.9 (0.02, 9.2)	>0.99	>0.99

Pooled dataset includes evaluable patients from the current FIGHT-207 study (n=13) and the open-label, phase 2 FIGHT-201 study (n=149) with urothelial carcinoma and best overall responses reported.
* Fisher's exact test, two-sided, for count data.
† False discovery rate correction for multiple testing.

Supplementary Table 8. Principal investigators and institutions participating in FIGHT-207

Principal Investigator	Institution	Institution Country
Eugene Ahn	Cancer Treatment Centers of America	USA
Myung-Ju Ahn	Samsung Medical Center	Republic of Korea
Jordi Rodon	The University of Texas MD Anderson Cancer Center	USA
Jerome Alexandre	Hospital Cochin Cancerologie	France
Salwan Al-Mutar	UT Southwestern Medical Center	USA
Daisuke Aoki	Keio University Hospital	Japan
Andrea Ardizzoni	L'Azienda Ospedaliero-Universitaria di Bologna Policlinico S. Orsola – Malpighi	Italy
Hendrik-Tobias Arkenau	Sarah Cannon Research Institute	UK
Gil Bar-Sela	Ha Emek Medical Center	Israel
Stefan Boeck	University Hospital Grosshadern Munich	Germany
Esma Bouzid	Centre Antoine Laccassagne	France
John Bridgewater	University College London Hospitals NHS Foundation Trust	UK
Silvia Damian	Comitato Etico Fondazione IRCCS Istituto Nazionale dei Tumori Milano	Italy
Emanuela Dellaquila	I.F.O. Istituto Nazionale Tumori Regina Elena	Italy
Muhammad Furqan	University of Iowa Hospital and Clinics	USA
Masayuki Furukawa	National Hospital Organization Kyushu Cancer Center	Japan
Ana Galeote Miguel	Hospital Regional Universitario de Malaga	Spain
Javier Garcia Corbacho	Hospital Clinic Barcelona Main	Spain

Jesus Garcia-Donas Jimenez	Centro Integral Oncologico Clara Campal	Spain
Carlos Alberto Gomez-Roca	Universitaire du Cancer de Toulouse Institut Claudius Regaud IUCT Oncopole	France
Lipika Goyal	Massachusetts General Hospital	USA
Vikas Gupta	Comprehensive Cancer Center of Nevada	USA
Alice Hervieu	Centre Georges Francois Leclerc	France
Seock-Ah Im	Seoul National University Hospital	Republic of Korea
Hiroo Imai	Tohoku University Hospital	Japan
Antoine Italiano	Institut Bergonie	France
Oscar Jose Juan Vidal	Hospital Universitari i Politecnic La Fe	Spain
Santosh Kesari	Providence St. Joseph Hospital Orange Center for Cancer Prevention and Treatment	USA
Fachyi Lee	Chao Family Comprehensive Cancer Center University of California, Irvine	USA
Amit Mahipal	Mayo Clinic	USA
Marc Matrana	Ochsner Clinic Foundation, Ochsner Cancer Institute	USA
Timothy McCarthy	Virginia Cancer Specialists, PC	USA
Niharika Mettu	Duke University Hospital	USA
Hironobu Minami	Kobe University Hospital	Japan
Mariano Ponz-Sarvise	Clinica Universidad de Navarra	Spain
Fernando Rivera Herrero	Hospital Universitario Marques de Valdecilla	Spain

Caio Rocha Lima	Wake Forest University, Center for Dermatology Research	USA
Clorinda Schettino	Istituto Nazionale Tumori IRCCS Fondazione Pascale	Italy
Richard Siegel	Illinois Cancer Specialists	USA
Sung Hoon Sim	National Cancer Center	Republic of Korea
Iben Spanggaard	Rigshospitalet	Denmark
Salomon Stemmer	Rabin Medical Center – Beilinson Hospital	Israel
Shinji Takeuchi	Kanazawa University Hospital	Japan
Makoto Ueno	Kanagawa Cancer Center	Japan
Susanna Ulahannan	Oklahoma University Medical Center	USA
Daniel Vaena	The West Clinic PC	USA
Maria Vieito	Hospital General Universitario Vall d'Hebron	Spain
Tomoya Yokota	Shizuoka Cancer Center	Japan

Supplementary Figure 1. Comparison of *FGFR* Alterations (A) and Co-Alterations (B) Detected in Patients With Both Tissue (Archival or Baseline) and ctDNA (Baseline) NGS Samples (Cohorts A, B, and C; N=89). Key variant genes and variant gene pathways detected in patients with NGS analysis of both archival or baseline tissue samples and baseline ctDNA samples are shown. Concordant gene alterations were detected in both ctDNA and tissue from the same patient. Discordant gene alterations (ie, detection of *FGFR2* fusions with different partners in ctDNA and tissue from the same patient) were detected in a minority of patient samples.   MAPK pathway includes *KRAS*, *NRAS*, and *BRAF*. ctDNA, circulating tumor DNA; FGFR, fibroblast growth factor receptor; NGS, next-generation sequencing.

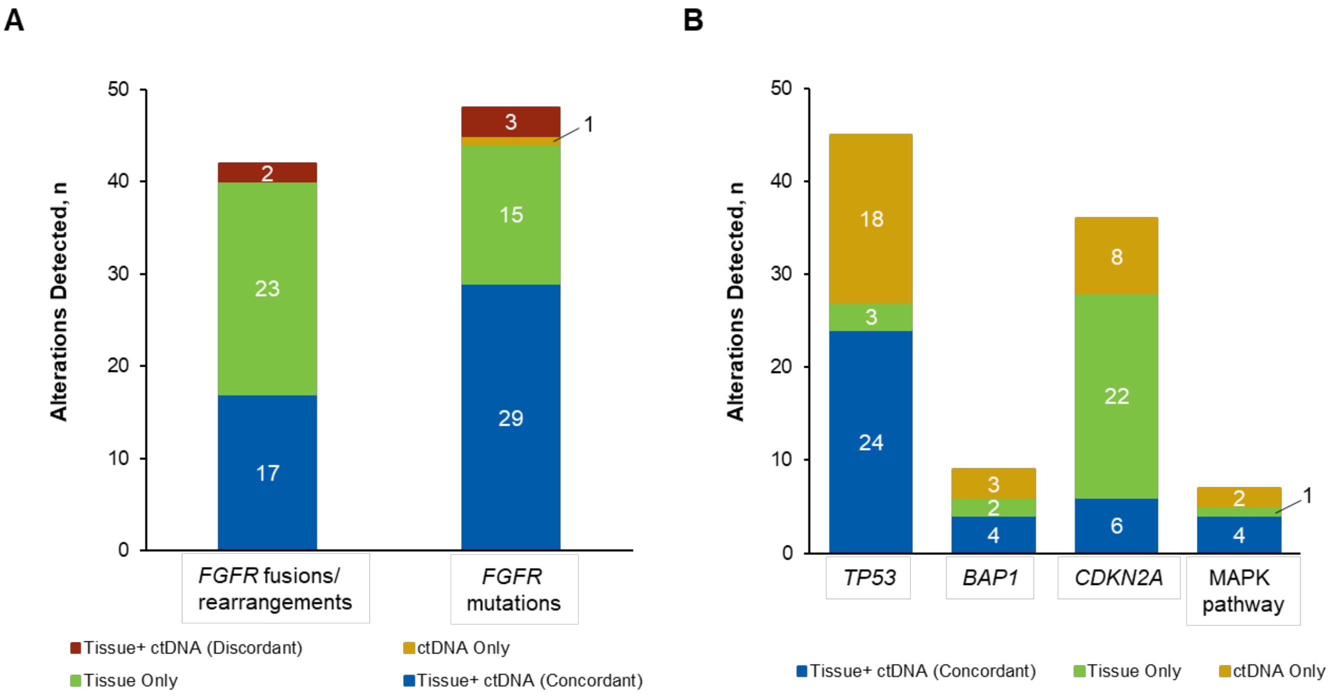

Supplement: Supplementary file 1 — Supplementary Tables 1–8 and Fig. 1. [file 41591_2024_2934_MOESM1_ESM.pdf]
