# Supplementary material for: Pemigatinib in previously treated solid tumors with activating FGFR1–FGFR3 alterations: phase 2 FIGHT-207 basket trial
Source: Nat Med. 2024 May 6;30(6):1645–54. doi: 10.1038/s41591-024-02934-7 (PMC11186762; doi:10.1038/s41591-024-02934-7)
Supplement: Supplementary file 2 — Reporting Summary [file 41591_2024_2934_MOESM2_ESM.pdf]

nature portfolio

Corresponding author(s): Lipika Goyal; Jordi Rodon

Last updated by author(s): March 14, 2024

Reporting Summary

Nature Portfolio wishes to improve the reproducibility of the work that we publish. This form provides structure for consistency and transparency in reporting. For further information on Nature Portfolio policies, see our Editorial Policies and the Editorial Policy Checklist.

Statistics

For all statistical analyses, confirm that the following items are present in the figure legend, table legend, main text, or Methods section.

n/a	Confirmed	
☐	☒	The exact sample size (*n*) for each experimental group/condition, given as a discrete number and unit of measurement
☐	☒	A statement on whether measurements were taken from distinct samples or whether the same sample was measured repeatedly
☐	☒	The statistical test(s) used AND whether they are one- or two-sided<br>*Only common tests should be described solely by name; describe more complex techniques in the Methods section.*
☒	☐	A description of all covariates tested
☐	☒	A description of any assumptions or corrections, such as tests of normality and adjustment for multiple comparisons
☐	☒	A full description of the statistical parameters including central tendency (e.g. means) or other basic estimates (e.g. regression coefficient) AND variation (e.g. standard deviation) or associated estimates of uncertainty (e.g. confidence intervals)
☐	☒	For null hypothesis testing, the test statistic (e.g. *F*, *t*, *r*) with confidence intervals, effect sizes, degrees of freedom and *P* value noted<br>*Give P values as exact values whenever suitable.*
☒	☐	For Bayesian analysis, information on the choice of priors and Markov chain Monte Carlo settings
☒	☐	For hierarchical and complex designs, identification of the appropriate level for tests and full reporting of outcomes
☒	☐	Estimates of effect sizes (e.g. Cohen's *d*, Pearson's *r*), indicating how they were calculated

Our web collection on statistics for biologists contains articles on many of the points above.

Software and code

Policy information about availability of computer code

Data collection	Clinical data were entered into electronic case report form per the protocol. Data were managed by an electronic data capture system.
Data analysis	Data analyses were performed according to the statistical analysis plan using SAS v9 or higher. Translational data analyses were performed in R 4.1.1

For manuscripts utilizing custom algorithms or software that are central to the research but not yet described in published literature, software must be made available to editors and reviewers. We strongly encourage code deposition in a community repository (e.g. GitHub). See the Nature Portfolio guidelines for submitting code & software for further information.

Data

Policy information about availability of data

All manuscripts must include a data availability statement. This statement should provide the following information, where applicable:

- Accession codes, unique identifiers, or web links for publicly available datasets
- A description of any restrictions on data availability
- For clinical datasets or third party data, please ensure that the statement adheres to our policy

Incyte Corporation (Wilmington, DE, USA) is committed to data sharing that advances science and medicine while protecting patient privacy. The study protocol with confidential information redacted is provided in the Supplementary Information. Qualified external scientific researchers may request anonymized datasets owned by Incyte for the purpose of conducting legitimate scientific research. Researchers may request anonymized datasets from any interventional study (except

Phase 1 studies) for which the product and indication have been approved on or after 1 January 2020 in at least one major market (eg, US, EU, JPN). Data will be available for request after the primary publication or 2 years after the study has ended. Information on Incyte's clinical trial data sharing policy and instructions for submitting clinical trial data requests are available at: https://www.incyte.com/Portals/0/Assets/Compliance%20and%20Transparency/clinical-trial-data-sharing.pdf?ver=2020-05-21-132838-960

Research involving human participants, their data, or biological material

Policy information about studies with human participants or human data. See also policy information about sex, gender (identity/presentation), and sexual orientation and race, ethnicity and racism.

Reporting on sex and gender	Sex and/or gender were not considered in the study design or statistical analysis plan because fibroblast growth factor receptor (FGFR) alterations across histologies have not been shown to consistently predominate in one sex (Murugesan, et al. 2022). Patients were recruited into the study irrespective of sex or gender. The sex of the patients was self-reported and gender was not collected. No sex- or gender-based analyses were performed.
Reporting on race, ethnicity, or other socially relevant groupings	Self-reported race and ethnicity data were collected
Population characteristics	Patients had previously treated, advanced solid tumors with alterations in FGFR genes. Median age among efficacy-evaluable patients was 62 years; 57% were women, 69% were White, and 23% were Asian. The most commonly represented histologies were cholangiocarcinoma (16%), urothelial tract/bladder cancer (11%), and glioblastoma (9%). Efficacy-evaluable patients were divided into 3 cohorts: FGFR fusions/rearrangements (cohort A; n=49), FGFR actionable single nucleotide variants (cohort B; n=32), FGFR kinase domain mutations and variants of unknown significance (cohort C; n=26). Approximately half of the efficacy evaluable population received prior radiation (45%) and prior surgery for cancer (57%). Nearly all patients received prior systemic therapy (88%).
Recruitment	Eligible patients were ≥18 years old with a histologically or cytologically confirmed advanced/metastatic or surgically unresectable solid tumor and radiographically measurable disease per RECIST v1.1 or RANO. Patients were required to have a documented FGFR1–3 mutation or fusion/rearrangement, disease progression after ≥1 line of prior systemic therapy, no therapy available likely to provide clinical benefit, Eastern Cooperative Oncology Group performance status ≤2, a baseline tumor specimen, and willingness to avoid pregnancy or fathering children. Key exclusion criteria were prior treatment with a selective FGFR inhibitor, clinically significant corneal or retinal disorder, evidence of ectopic mineralization or calcification, and protocol-defined abnormal laboratory values. A full list of patient selection criteria are included in the Methods section.<br><br>The study was mainly conducted with sites that had previously worked in other pemigatinib studies in patients with cholangiocarcinoma and bladder cancer, which may explain the relatively high number of patients with these diseases in FIGHT-207. The protocol, however, had provision to cap certain tumor types including cholangiocarcinoma and bladder cancer, as well as FGFR1-3 alterations to allow representation of multiple tumor types and analysis being impacted by the overrepresentation of any individual tumor type.<br>Further, patients were enrolled by the study sites after molecular tumor board review and through referrals from peers. Referral letters detailing key inclusion criteria for the clinical trial were sent by Investigative sites to other departments within the study hospitals and to peers.
Ethics oversight	The study was performed in accordance with the International Council for Harmonisation Good Clinical Practice, the principles embodied by the Declaration of Helsinki, and local regulatory requirements. The study protocol was approved by the institutional review board of each study site before patient enrollment. All patients provided written informed consent prior to screening. A list of investigators and institutions participating in the study is provided in the Supplementary Information.

Note that full information on the approval of the study protocol must also be provided in the manuscript.

Field-specific reporting

Please select the one below that is the best fit for your research. If you are not sure, read the appropriate sections before making your selection.

☒ Life sciences ☐ Behavioural & social sciences ☐ Ecological, evolutionary & environmental sciences

For a reference copy of the document with all sections, see nature.com/documents/nr-reporting-summary-flat.pdf

Life sciences study design

All studies must disclose on these points even when the disclosure is negative.

Sample size	Approximately 60 and 90 patients were planned for cohort A and B respectively. Assuming objective response rates (ORRs) of 35% in cohort A and 30% in cohort B, respectively, 60 and 90 patients were needed to ensure ≥90% power to reject the null hypothesis of ORR ≤15% with a 1-sided test at the overall 0.025 level of significance. In cohort C, ≈20 patients were enrolled to provide ≥80% chance of observing at least 4 responders if the underlying ORR were 30%.
Data exclusions	There were 4 patients from whom FGFR alterations could not be centrally confirmed. Per the protocol, these patients were excluded from the efficacy analysis but included in the safety analysis.

Replication	No attempts were made to replicate the study findings as this was an exploratory, phase 2 study. Extensive demographic and clinical characteristics of enrolled patients are provided to support comparisons between this population with patients enrolled in other studies or included in other datasets.
Randomization	No randomization was undertaken for this open-label study. This study is a single-arm, open label study where all participants received the same treatment regimens. The cohort was assigned based on the FGFR mutations or translocations, and no comparisons were made between cohorts. Therefore, randomization was not needed.<br>This is not relevant to our study. This study is a single-arm, open label study where all participants will receive the same treatment regimens. The cohort was assigned based on the FGFR mutations or translocations, and no comparisons will be made between cohorts. Therefore, randomization is not needed.
Blinding	This study was designed to be open-label; therefore, no blinding was performed.

Reporting for specific materials, systems and methods

We require information from authors about some types of materials, experimental systems and methods used in many studies. Here, indicate whether each material, system or method listed is relevant to your study. If you are not sure if a list item applies to your research, read the appropriate section before selecting a response.

Materials & experimental systems

n/a	Involved in the study
☒	☐ Antibodies
☒	☐ Eukaryotic cell lines
☒	☐ Palaeontology and archaeology
☒	☐ Animals and other organisms
☐	☒ Clinical data
☒	☐ Dual use research of concern
☒	☐ Plants

Methods

n/a	Involved in the study
☒	☐ ChIP-seq
☒	☐ Flow cytometry
☐	☒ MRI-based neuroimaging

Clinical data

Policy information about clinical studies

All manuscripts should comply with the ICMJE guidelines for publication of clinical research and a completed CONSORT checklist must be included with all submissions.

Clinical trial registration	ClinicalTrials.gov Identifier: NCT03822117
Study protocol	The full study protocol is provided as Supplementary Information. Some confidential information is redacted.
Data collection	The study was conducted at 48 hospitals or academic centers in 10 countries (Denmark, France, Germany, Israel, Italy, Japan, Republic of Korea, Spain, United Kingdom, United States). A full list of investigators and study sites is provided in the Supplementary Information. Patients were enrolled between October 17, 2019 and July 12, 2021. The study was completed on March 29, 2022.
Outcomes	The primary endpoints were ORRs in cohorts A and B as determined by an independent review committee (IRC). ORR was defined as the percentage of patients who achieved complete response or partial response per RECIST v1.1 or RANO. Disease was assessed by computed tomography or magnetic resonance imaging (MRI) at baseline, every 3 cycles, and end of treatment. Secondary endpoints were IRC-assessed progression-free survival (time from first dose to progressive disease or death, whichever is first) in cohorts A and B, respectively, duration of response (time from the first assessment of complete response or partial response until progressive disease or death, whichever is first) in cohorts A and B, respectively, overall survival (time from first dose to death) in cohorts A and B, respectively, and safety and tolerability as assessed by the incidence and severity of treatment-emergent adverse events (AEs) and treatment-related AEs according to the National Cancer Institute Common Terminology Criteria for Adverse Events v5.0.

Plants

Seed stocks	Plants were not used in this study
Novel plant genotypes	Plants were not used in this study
Authentication	Plants were not used in this study

Magnetic resonance imaging

Experimental design

Design type	Whole brain MRI was used as an imaging tool to assess tumor responses in patients with central nervous system (CNS) tumors in FIGHT-207.
Design specifications	Sites performed MRIs for patients with CNS tumors in accordance with the sponsor-defined imaging charter. The sponsor did not standardize MRIs across sites. Tumor responses were assessed by independent central radiologic review according to RANO criteria. Briefly, sites sent deidentified images to the independent reader on CDs or DVDs in DICOM format. The independent reader checked the images for technical quality (e.g., absence of patient motion or artifact, presence of whole anatomical region and all timepoints), compliance with imaging guidelines, and consistent imaging across multiple timepoints. The independent reader then reviewed quality-checked images.
Behavioral performance measures	Behavioral performance was not assessed in FIGHT-207

Acquisition

Imaging type(s)	Brain tumor imaging protocol
Field strength	1.5T, 3T
Sequence & imaging parameters	• Sagittal/axial 3D T1w pre-contrast<br>• Axial 2D FLAIR (TSE)<br>• Axial 2D DWI<br>• Axial 2D T2w (TSE)<br>• Sagittal/axial 3D T1w post-contrast
Area of acquisition	Whole brain

Diffusion MRI ☐ Used ☒ Not used

Preprocessing

Preprocessing software	Preprocessing, normalization, noise and artifact removal, and volume censoring were performed by sites. Details were not collected by the sponsor.
Normalization	Preprocessing, normalization, noise and artifact removal, and volume censoring were performed by sites. Details were not collected by the sponsor.
Normalization template	Preprocessing, normalization, noise and artifact removal, and volume censoring were performed by sites. Details were not collected by the sponsor.
Noise and artifact removal	Preprocessing, normalization, noise and artifact removal, and volume censoring were performed by sites. Details were not collected by the sponsor.
Volume censoring	Preprocessing, normalization, noise and artifact removal, and volume censoring were performed by sites. Details were not collected by the sponsor.

Statistical modeling & inference

Model type and settings	Statistical modeling and inference was not performed
Effect(s) tested	Statistical modeling and inference was not performed

Specify type of analysis: ☐ Whole brain ☐ ROI-based ☐ Both

Statistic type for inference<br>(See Eklund et al. 2016)	Statistical modeling and inference was not performed
Correction	Statistical modeling and inference was not performed

Models & analysis

n/a	Involved in the study
☒	☐ Functional and/or effective connectivity
☒	☐ Graph analysis
☒	☐ Multivariate modeling or predictive analysis
